# Young Adults’ Belief in Genetic Determinism, and Knowledge and Attitudes towards Modern Genetics and Genomics: The PUGGS Questionnaire

**DOI:** 10.1371/journal.pone.0169808

**Published:** 2017-01-23

**Authors:** Rebecca Bruu Carver, Jérémy Castéra, Niklas Gericke, Neima Alice Menezes Evangelista, Charbel N. El-Hani

**Affiliations:** 1 Department of Communication, Norwegian Institute of Public Health, Oslo, Norway; 2 Aix-Marseille Université, EA4671 ADEF, ENS de Lyon, Aix-Marseille, France; 3 Department of Environmental and Life Sciences, Karlstad University, Karlstad, Sweden; 4 History, Philosophy, and Biology Teaching Lab, Institute of Biology, Federal University of Bahia, Salvador, Brazil; University of Rochester, UNITED STATES

## Abstract

In this paper we present the development and validation a comprehensive questionnaire to assess college students’ knowledge about modern genetics and genomics, their belief in genetic determinism, and their attitudes towards applications of modern genetics and genomic-based technologies. Written in everyday language with minimal jargon, the Public Understanding and Attitudes towards Genetics and Genomics (PUGGS) questionnaire is intended for use in research on science education and public understanding of science, as a means to investigate relationships between knowledge, determinism and attitudes about modern genetics, which are to date little understood. We developed a set of core ideas and initial items from reviewing the scientific literature on genetics and previous studies on public and student knowledge and attitudes about genetics. Seventeen international experts from different fields (*e*.*g*., genetics, education, philosophy of science) reviewed the initial items and their feedback was used to revise the questionnaire. We validated the questionnaire in two pilot tests with samples of university freshmen students. The final questionnaire contains 45 items, including both multiple choice and Likert scale response formats. Cronbach alpha showed good reliability for each section of the questionnaire. In conclusion, the PUGGS questionnaire is a reliable tool for investigating public understanding and attitudes towards modern genetics and genomic-based technologies.

## Introduction

The underlying motivation for developing the Public Understanding and Attitudes towards Genetics and Genomics (PUGGS) questionnaire is to facilitate investigation into the relationship between three key elements: belief in genetic determinism, knowledge of modern genetics, and attitudes towards modern genetics and genomic-based technologies. As we use the expression here, “modern genetics” encompasses advances in genetics and related areas over the past twenty years, including genomics and genomic-based technologies such as pharmacogenomics and new cancer therapies. Modern genetics and genomics adopt a more systemic understanding of genes and their functioning within cells than earlier genetics, conceiving environmental and epigenetic factors as playing an important role in the development of traits and diseases [1, 2], although there are some concerns that genetics is still too gene- and cell-centric [3–5]. Advances in genetics as well as genomics and genomic-based technologies are becoming increasingly relevant in all areas and layers of modern society, from prenatal testing, direct-to-consumer genetic tests, to personalized medicines, pharmacogenomics and new cancer therapies [6–9]. The demands on the public for genetic and genomic literacy are probably higher than they have ever been.

It is important to clarify from the very start how we understand the word ‘belief’ in our study. Belief is the state of mind in which a person thinks something to be the case, with or without there being convincing reasons that something is the case with factual certainty [10]. In science, convincing reasons are many times but not always based on empirical evidence. In other ways of knowing, convincing reasons may be different. It is also important to consider that belief and knowledge are not opposite concepts. Knowledge is the state of mind in which a person thinks something to be the case, with convincing reasons that something is the case with factual certainty. That is, knowledge is justified belief. When there are no convincing reasons, then we should talk only about belief.

Another concept that seems important to explain is ‘understanding’, which we take to mean making sense of a certain idea by connecting it with other ideas we already know, making it intelligible in this manner [11].

Research over the past twenty-five years has shown there is a small positive link between knowledge and attitudes in science in general [12, 13]. However, the relationship can also be negative, depending on the particular topic or domain of science and technology at hand [12, 13]. When it comes to genetics, Condit [14] has pointed out that “contrary to the assumption that ‘more education’ will result in more positive reactions to genetic research, the existing polls indicate that negative reactions to genetic technologies might not simply reflect a lack of education or understanding” (p. 812). Previous studies reveal a variety of trends: some have shown that people with higher levels of understanding also have more opposition towards genetic technologies, whereas other studies showed no or little relationship between genetic understanding and attitude [13].

At the same time, studies within genetics education and public understanding of genetics persistently show that the public and, more specifically students, have rather low knowledge of genetics. Genetic deterministic views about the nature of genes, behavior and biological traits have been found among students, teachers, textbooks, and the public [15–22]. Genetic determinism is the belief that genetic contributions to phenotypes are exclusively or at least much more important than the contributions of other factors such as epigenetic and environmental ones, even in the case of complex traits such as behaviors and personality [23].

Despite the low knowledge level, opinion polls indicate that the public are generally positive towards genetic applications where there are clear medical benefits, such as genetic testing for diagnosis and treatment of disease [14, 24, 25]. Could it be that deterministic understandings of genetics make people believe more in the power of genes and genetic technologies, assuming that genes are distinctive entities that can be modified and “fixed”? How do genetics knowledge and belief in genetic determinism interact with each other, and with attitudes toward genetic applications?

Overall, the relationship between determinism, knowledge and attitudes about genetics is unclear. Most of the previous studies have focused either on genetic literacy (knowledge), or on attitudes towards genetics, and often on narrow aspects of genetics or genomics. As far as we can tell, there are no questionnaire instruments currently available that, in a single instrument, address all the three issues: (i) belief in genetic determinism, (ii) knowledge of modern genetics and genomics, and (iii) attitudes towards modern genetics and genomic-based technologies. We have therefore developed the PUGGS questionnaire in order to facilitate further investigation into the relationships between knowledge, genetic determinism and attitudes towards modern genetics and genomics. We report here how we built and validated this questionnaire as a reliable tool to investigate those relationships. The PUGGS questionnaire can be used, for example, to find out to what extent the public knowledge reflects perspectives on genetic determinism, and whether this in turn influences their knowledge and attitudes towards applications of modern genetics and genomics.

### Theoretical background

#### Genetic determinism

Deterministic understandings of genetics typically focus on a one-to-one relationship between genes, proteins, functions, and traits, as if particular traits or diseases were generally related to a single gene. Genetic determinism is often implied in common expressions such as “the gene for intelligence”, and ignores the influence of environmental and epigenetic factors [1, 26]. Nevertheless, the expression “gene for” [2] has subtleties in use and interpretation that are often lost from sight. The expression “gene for” can be conceived as a shorthand expression for “a locus in which sequence variation causes a difference in phenotype, all other things being equal” [27, p. 298, citing 28]. However, there are many cases in which we cannot interpret phenotypic differences as traceable to a single locus, since a whole series of loci affect the normal functioning of the biochemical processes involved in the development of the trait—for instance, the biochemical pathways that lead to the synthesis of eye pigments. Moreover, epigenetic and environmental factors are as a rule involved in the development of phenotypes and phenotypic differences. If we consider, say, the textbook example of a “gene for blue eyes”, we will see that it corresponds, in fact, to a disjunction of alleles that can be responsible for a decrease of the pigmentation in the iris, and a disjunction is a logical expression, not a material entity to which a concept can refer [29]. This does not deny, however, the usefulness of the “gene for” concept: It helps us understand the results of a crossing between a brown-eyed father and a blue-eyed mother, and we can readily use pedigree analysis, accompanied by the apt simplification of assuming genes that determine the presence of brown or blue eyes.

What is at stake is that the deterministic gene is an instrumental concept that has predictive power and plays an important role in some explanations in genetics and molecular biology, but cannot be conflated with the molecular gene [30], which is a necessary but not sufficient condition for the development of many phenotypic traits [31]. There has been a tendency, however, for both scientists and lay people alike to overemphasize the importance of genes, applying deterministic thinking to conceive the role of genes in the development of complex multifactorial traits and disorders [15, 32], thus extending the deterministic interpretation beyond the domain in which it may be appropriate. Deterministic thinking has been used, for instance, in gene-centric explanations of complex phenomena like criminal behavior, political participation or sexual orientation (as discussed in [33]). This genetic essentialist belief may be problematic for our society since it engenders intolerant attitudes such as racism and prejudice against sexual orientation [16, 33–36].

**Fig 1:**
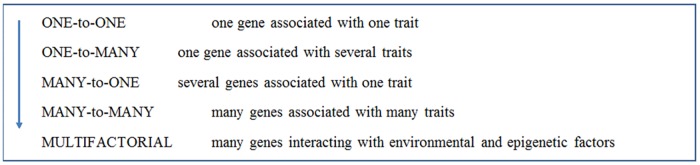
From deterministic to probabilistic understanding about genetics and genomics.

In the last two decades, however, research in genetics has evolved into genomics and our understanding of genes and genomes and how they relate to development, phenotypic traits, cell physiology, among other features, has radically changed [29, 37–40]. It seems to be under way a general shift within the scientific community from a more deterministic to a more probabilistic understanding of the relationship between genes and traits (Fig 1). Advances in genetics and genomics have made it clear that gene action and function should be conceived as embedded into multiple hierarchical levels, in which complex networks of interactions between components are the rule [41]. Consequently, the probabilistic understanding of the structure, dynamics and functions of genes demands that they are located in complex informational networks and pathways [41].

Genetic science now tells us that, despite the usefulness of the deterministic gene as an instrumental concept in some explanatory tasks, in realistic terms it is not possible for any trait—even so-called “single-gene disorders”–to be determined by genes only, due to the influence of epigenetic and environmental factors [1, 2, 42]. There can be many genes associated with one trait, or many different traits associated with the same genes, which in turn are affected by a myriad of environmental factors. As shown in Fig 1, as we move from a “one-to-one” (deterministic) to a “multifactorial” (probabilistic) model of the relation between genes and traits, environmental and epigenetic factors are seen as playing a more important role in the development of traits and diseases. In the probabilistic model, genes are embedded in the context of an internal and external environment, with due attention to the fact that many genetic and epigenetic factors interact with one another, and, also, that genetic and environmental factors often interact non-additively, so that genes show different expressivity and penetrance depending on the influence of environmental factors [1, 4, 5].

#### Public knowledge about modern genetics and genomics

Despite the progress of genetics as a research field, the developments in scientific understanding seem not to have reached the public: genetic deterministic beliefs still prevail to a great extent, according to the literature [15, 16, 26, 32, 33, 43–45]. Previous studies examining public knowledge of genetics generally indicate that such knowledge is low [20, 21, 46, 47]. In particular, people have difficulty understanding the mechanisms behind genetic risk [48], as well as the notion that genetic and environmental factors interact [9, 49, 50]. Most previous studies of the public understanding of genetics have been qualitative focus groups, case or interview studies [20, 21, 46, 49], as well as a few large-scale survey or questionnaire-based studies [47, 48].

Studies of mass media portrayals of genetics also generally indicate a predominant discourse of determinism [15, 32, 45]. Nelkin and Lindee [15] have advocated that public discourse on genetics is plagued by genetic determinism in such a way that human beings equate “all their social, historical, and moral complexity, with their genes” (p. 2). They have argued that determinism, or ‘genetic essentialism’ as they call it, is not simply a result of misunderstanding or simplification of science, but could be anchored in deep beliefs and social phenomena. As an example of such deep beliefs, Parrott et al. found that some people believe God plays an important role in how genes are expressed and impact health [51]. They believe, for example, that a higher power protects human genes from the effects of people’s unhealthy behaviours. This shows how predetermined views of biological outcomes may be deeply rooted in religious beliefs.

Studies on how genetics is taught in schools generally indicate that the predominant mode of genetics instruction, as well as the content of genetics textbooks, prime students to think deterministically [17, 43, 44, 52, 53]. It has been also shown that when students learn or talk about molecular genetics they tend to draw on more deterministic Mendelian explanations [54, 55]. In particular, according to Dougherty [44], “many students view phenotypes through the lens of Mendelian inheritance and fail to appreciate that most human traits are the product of polygenic expression modulated by the environment” (p.7). In addition, very few students have heard of newer concepts within genetics such as those relating to epigenetics [22]. Moreover, it has been shown that a significant part of primary and secondary school teachers (in Biology and in language) from different countries have strong genetic deterministic beliefs about some human personality traits [16].

Students in high school find genetics particularly challenging to learn because it requires reasoning across multiple organizational levels: between genes, proteins, cells, tissues, and organs [56, 57]. Students find it difficult to understand how mechanisms and interactions at the molecular (genes, proteins) and micro-levels (cells) bring about effects at the macro-level (organism, population) [56]. Moreover, they find it difficult to understand the mechanisms of gene expression, and how environmental factors interact with genes [22]. Within public health, genetic determinism is thought to have a negative impact on people’s understanding of health and disease [32, 33, 43, 51]. Genetic deterministic views can, for example, lead people to devalue the role of environmental or experiential factors in a number of traits such as mental illness, cancer, obesity, diabetes, which in turn can hamper efforts to prevent diseases [51]. In general, it is believed, thus, that genetic determinism poses a threat to genetic and health literacy.

#### Public attitudes towards modern genetics and genomics

The concept of attitudes has been borrowed from the social psychology field into other areas such as public understanding of science and science education research. Fazio [58] gives a general definition of the concept: “An attitude is viewed as an association in memory between a given object and one's evaluation of that object. This definition implies that the strength of an attitude, like any construct based on associative learning, can vary”. Fazio’s model assumes that attitudes are important factors to predict behaviors. Even if the level of consistency of attitudes and behaviors is still discussed among experts, some empirical studies have shown a strong relationship between them [e.g. 59, 60].

Whilst public knowledge about modern genetics seems to be low, studies on public attitudes towards genetics and gene technologies indicate that the public are generally positive towards genetic applications, particularly if there are clear medical benefits [14, 24, 25]. There are, however, some specific genetic technologies that the public are more negative towards, such as cloning of human cells, human embryo research, and using genetic diagnoses to decide whether or not to continue a pregnancy [12, 24, 25]. People tend to be ambivalent towards genetically modified foods [14, 24, 25]. On the whole, we can say that people’s attitudes tend to be more favorable for uses of genetics that maintain a perceived “natural order” and more unfavorable for uses that are perceived to change that order.

Very few studies have investigated the possible links between attitudes towards genetics and belief in genetic determinism and/or genetic knowledge. A study about attitudes towards genetic testing among the Dutch public found no association between people’s attitudes towards genetic testing and their level of genetic knowledge or belief in genetic determinism [61]. A study by Rose et al. [62], in turn, found that in a U.S. sample, after adjustment for sociodemographic characteristics and family cancer history, higher knowledge about genetic testing for cancer risk was correlated with more positive attitudes towards genetic testing. However, this study did not include genetic determinism as a variable in their questionnaire. It is clear, thus, that more studies are needed, considering that belief in genetic determinism may be an intervening factor in the relation between knowledge about genetics and attitudes towards genetic applications.

### Previous questionnaires

Within genetics education, several questionnaire instruments have been developed for testing students’ genetic literacy—particularly among university students. Smith, Wood and Knight [63] developed a Genetics Concept Assessment for undergraduate science and non-science students, with 25 multiple choice questions. The questions tested fairly detailed knowledge, such as about genetic transmission and inheritance of various traits and diseases. Similarly, Bowling et al. [64] developed the Genetic Literacy Assessment Instrument (GLAI), which includes 31 multiple choice items addressing 17 concepts and is targeted towards non-science undergraduates. Their items are based on six core areas of genetics knowledge that are deemed essential to genetic literacy: nature of the genetic material, transmission, gene expression, gene regulation, evolution, and genetics and society. To our knowledge, no previous questionnaire has specifically measured public knowledge about genomics, but some concepts in genomics related to gene regulation and expression have been included in these previous genetic literacy assessments.

Scales for measuring belief in genetic determinism have been developed, for the most part, in studies relating genetic determinism to sociopolitical issues such as social prejudice and psychology, racism, sexism and genetic discrimination [16, 34–36]. The items in such scales are often emotionally loaded; for example, in a study about genetic discrimination and racism [34] the item “Racial differences in academic ability are caused by genetics” may reflect more a personal and political perspective about racism than belief in genetic determinism only. Generally speaking, we judged that these previous determinism items were not directly relevant to our questionnaire about modern genetics and genomics, and, therefore, we devised new items for the PUGGS questionnaire.

The majority of determinism items have been measured using a Likert scale (for example, from 1 = strongly disagree to 5 = strongly agree). However, an Australian study by Molster et al. [48] used a different approach. They developed a questionnaire about knowledge of human genetics and health for the general public, comprising a set of true/false/don’t know questions on topics such as genetic risk, basic genetic concepts, inheritance, and determinism. Rather than using Likert scale questions, they asked respondents to classify a range of health conditions, such as eye colour, cystic fibrosis and heart disease, as being caused by genetics, the environment, or a mix of both genetic and environmental factors. Each condition had a correct or incorrect answer, depending on whether it was a wholly genetic, a multifactorial or a wholly environmental condition.

In turn, most of the studies in public attitudes towards genetics have been conducted using large opinion polls looking at biotechnology in general, mostly in Europe and the U.S. [65–67], but there were also smaller-scale questionnaire-based studies looking at specific topics such as genetic testing [61, 62, 68–70]. Other small-scale studies have looked at attitudes towards genomics more generally, including topics such as pre-implantation genetic diagnosis, human cloning, and gene therapy [13, 24]. These studies tend to use Likert scales, presenting statements about various issues and asking respondents the extent with which they agree or disagree with each statement.

Whilst there are clearly many questionnaires already available, each looking at specific issues or areas of modern genetics and its applications, some testing knowledge and others testing attitudes, there is a need for a coherent instrument addressing multiple purposes. First, there is a need to develop an instrument to encompass both knowledge and attitudes about modern genetics. In that way it will be possible to investigate the still-unanswered question regarding the relationships between knowledge in genetics and attitudes towards genetic applications. Second, belief in genetic determinism has only in limited ways been included in previous instruments, and mostly in the context of socio-political and ethical issues such as racism and sexism. Therefore, we aimed at developing an instrument that, in a more context-independent way, investigates the level of genetic deterministic beliefs. Third, modern genetics and genomics have changed rapidly during the last decade, and, thus, there is a need for an instrument that covers a range of topics that have recently become quite relevant, such as, for instance, epigenetics. Based on these three gaps we have developed the PUGGS questionnaire in order to make it possible to inquire, with the same instrument, if there are any relations between young adults’ knowledge of modern genetics and genomics, their attitudes towards genetic applications, and their beliefs in genetic determinism.

The questionnaire was found to have high content validity and good internal consistency, which means that it can be used to give a reliable measure of belief in genetic determinism, knowledge of modern genetics and genomics, and attitudes towards applications derived from these fields.

## Materials and Methods

The research project has been approved by the Committee for Ethics in Research from Federal University of Bahia Nursing School, no. 1.023.782. This section describes how the questionnaire was developed and refined in response to several measures of content validity and internal consistency.

**Fig 2:**
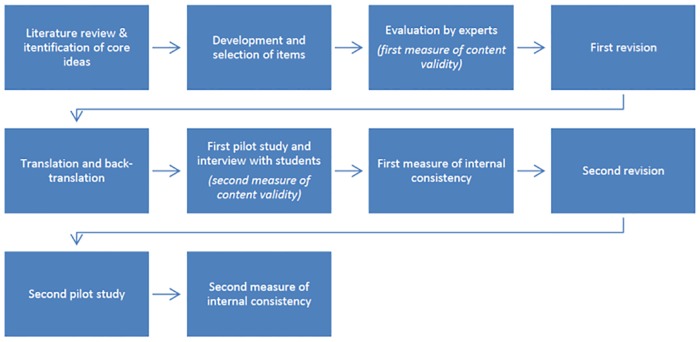
The process of the PUGGS questionnaire development.

The overall process of developing the PUGGS questionnaire involved the following main steps: item development, validation, and pilot testing. For the validation, we tested for content validity and internal consistency (reliability). The questionnaire was revised two times and piloted two times in response to the results of the validation. It was developed in English, translated into Portuguese, and thereafter validated with back-translation, since the pilot testing involved Brazilian students. The steps of the questionnaire development are shown in Fig 2.

### Item development

The PUGGS questionnaire consists of five sections: (1) Background information, (2) Belief in genetic determinism, (3) Knowledge about gene-environment interaction (which can also provide information related to genetic determinism), (4) Knowledge about modern genetics and genomics, and (5) Attitudes towards applications of modern genetics and genomics (to view the whole questionnaire, please see the S1 Table). The opening section on background information includes questions about respondents’ age, gender, education, religiousness, and experience with genetics. The overall themes and topics within modern genetics and genomics were chosen on the basis of what is currently considered relevant for genetic and genomic literacy [8, 9, 64, 71]. The questionnaire is focused on the human case, since the students are prompted to consider human traits, like schizophrenia or addiction.

#### Identification of core ideas and topics

In the first step of the process, we identified a set of core ideas about genetics and genomics. Our set of core ideas covers two main knowledge areas: gene-environment interaction (section three of the questionnaire), and knowledge about modern genetics and genomics (section four). These core ideas are like “knowledge benchmarks” that the science education and genetics communities consider important for high school education and general public knowledge [64, 71]. We searched and identified core ideas from the literature about genetics literacy and genetics education, in addition to using our own expertise within genetics and genetics education. A complete list of the core ideas with descriptions, corresponding items, and sources can be found in the S2 Table.

The core ideas about gene-environment interaction (Section 3) are about whether and how genes and environment interact in the development of phenotypic traits, which can in turn influence their degree of commitment to genetic deterministic thinking. They reflect the transition from a deterministic to a probabilistic understanding in genetics, as shown in Fig 1. To identify the core ideas for this section we consulted literature on genetics, epigenetics, philosophy, psychiatry, and genetics education [1, 2, 42, 72, 73].

The core ideas in modern genetics and genomics (Section 4) are divided into three sub-areas: (1) characteristics of the genome, (2) gene function and expression, and (3) epigenetics. These sub-areas represent key conceptual domains, currently deemed essential to genetics literacy [64, 71], as well as newly emerging topics such as epigenetics, which may or may not yet be a part of high school or university genetics curricula.

To define the core ideas, we drew heavily on several curriculum benchmarks in genetics that outline what high school students ought to know and what content should be taught [71, 74], on literature about the current status of genetics education [7, 44, 52, 75], on developments within the scientific and philosophical literature [72, 73], and on genetics textbooks [76]. Please see S2 Table for a full description of the core ideas.

The attitude section of the questionnaire (Section 5) covers four topics within modern genetic and genomic-based technologies: *gene therapy; genetic testing; prenatal genetic diagnosis; personalized medicine and pharmacogenomics*. These topics were chosen on the basis of what is considered important for the public to know about such technologies now and in the near future [8, 9]. Some of these genetic applications are already in use or are fairly well-known, such as genetic testing and prenatal genetic testing. Others are currently emerging and are likely to become relevant for people’s lives in the near future, such as gene therapy, personalized medicine and pharmacogenomics.

#### Development and selection of questionnaire items

In the next step, we developed a set of questions (items) that would test the core ideas. Where it was possible to relate our core ideas and topics to existing items in other questionnaire instruments, we gave priority to these items since they had already been validated and tested. Hence, most of the items in our questionnaire are based on items that have been used in previous studies, but we have modified them in order to match our core ideas. We also used the combined expertise in genetics and genetics education of the authors to help select and refine the items. Some of the items were also developed by the authors themselves.

Section two (belief in genetic determinism) consists of a task in which students were asked to indicate the relative importance of genes or environments for various traits presented in a table (herein called “the table of traits”). For building the table of traits task, we browsed several high school biology textbooks published in Brazil, which were submitted by publishing companies to a Brazilian National Program that distributes textbooks for public high schools across the country. We searched these textbooks to find examples of traits and diseases that are commonly mentioned in genetics education, since we wanted respondents to have heard of them. The table of traits task was designed with a Likert-scale response format, from “only environmental differences contribute to the traits” to “only genetic differences contribute to the traits”. The answers were originally scored on a ten-point scale, but later reduced to a five-point scale in the revision process (see section 4.1.1. above). We assume answers to this task are based more on beliefs about genetic determinism than on knowledge.

Section three (knowledge about gene-environment interaction) consists of a set of True/False/Don’t know items designed to measure deterministic understandings of genes and traits (more knowledge about gene-environment interaction indicating less determinism, and vice-versa). We included a “Don’t know” option in order to uncover topics or concepts that respondents potentially have not heard of before. We consulted previous determinism scales [16, 48, 51, 61, 77], but found that most of these previous items were either too general (e.g., “When people know their genetic make-up they will not be able to lead their own lives”) [61] or were too closely connected to sociopolitical issues such as racism, or were based on specific topics that students may or may not have much prior knowledge about. We therefore drew on ideas from these previous items, but made them less topic-specific and more closely related to our core ideas (focusing on the transition between deterministic and probabilistic understandings of genes and traits).

The items about modern genetics and genomics (Section 3) were largely based on items from previous questionnaires [63, 64] as well as curriculum benchmarks in genetics education [71, 74]. We also consulted the scientific literature on genetics and genomics [9, 72, 73], especially for the items on the genome and epigenetics, since there were not many previous items to draw from.

To develop the attitude items, we reviewed items used in previous questionnaires about attitudes towards genetics and gene technologies [13, 24, 61, 68–70]. We used a four-point Likert scale response format (strongly disagree, disagree, agree and strongly agree). In the first pilot we also included a “Don’t know” option, to see if some of the items were too difficult, but we removed it after the pilot studies.

Our initial questionnaire, used in the first pilot, had 51 items and 20 core ideas (See the S3 Table).

### Content validity

We conducted two measurements of content validity: Firstly, the initial questionnaire was evaluated by a group of experts from different fields, and consequently revised. Secondly, the revised questionnaire was piloted to 207 first year university students and 19 of them were interviewed about the comprehensibility of the questions. Their responses were taken into consideration in a second revision of the questionnaire.

#### Evaluation by experts

To test the content validity of the core ideas and items, we invited international experts from a range of fields related to genetics to evaluate the initial questionnaire and the core ideas. The experts were personally invited by email; thirty-two were invited and seventeen responded and evaluated the questionnaire. Of the seventeen experts who responded, four were working as biologists or geneticists, eight were working in genetics education, four in the field of sociology of biology, and two in the history and philosophy of genetics (one of them was both geneticist and educator). In addition, four of them had extensive expertise in designing assessment instruments in science education.

The experts were given a feedback sheet that they were requested to fill in, which contained the questionnaire items and the core ideas to which they were related. For the core ideas we asked: “Please indicate if you have any comments to the core ideas, for instance if you think some are unnecessary, if some should be altered, or if other core ideas related to modern genetics and genomics are missing.” For the section on Background information they were asked to comment on whether they thought some of the questions in the section should be changed in any way, and why. For the table of traits task we asked them to provide any helpful comments on the table as a whole. For the knowledge items on gene-environment interaction and on modern genetics and genomics they were asked to answer the following three questions with “yes”, “no” or “maybe” for each item: (1) Does the question test the core idea?; (2) Is this a quality question?; (3) Is it redundant? For the attitude items they were asked to answer with “yes”, “no” or “maybe” whether or not each item adequately probed for positive or negative attitudes towards the topic in question. They were also asked to provide any useful comments to any aspects of the questionnaire as a whole (for example, suggestions for improvements in wording or content).

In the first revision of the PUGGS questionnaire, the experts’ comments were analyzed for themes, for example, if several of the experts made similar comments to a particular item, this was especially considered. Also, items with a lot of negative feedback were particularly taken into account in the revision process, as well as the experts’ corresponding suggestions for improvements. Many of the experts provided useful comments on how the items could be improved, and some also suggested new items. The entire questionnaire and the core ideas were altered in the first revision process.

#### Interviews with students

During the first pilot study, the questionnaire was administered to seven different classes on different occasions. At each of these sessions, after completing the questionnaire, two or three students were asked to participate in a short 3–5 minutes interview about their comprehension of the questionnaire items. We asked them three questions: 1) Did you find any of the questions difficult to understand?; 2) Why is the question difficult?; 3) Are there any words you didn’t understand?

### Internal consistency

Internal consistency (also referred to as internal reliability) is a measure of the strength of items that occur together within respective sections or themes, to see, for example, if there is a correlation between different items that test the same idea [78]. Internal consistency can be quantified as a coefficient, such as Cronbach alpha. A satisfactory coefficient is 0.7 or greater, although coefficients of 0.6 or greater are acceptable for newly created scales [79].

The internal consistency of the PUGGS questionnaire was measured in two pilot tests. The first pilot test was conducted following the first revision in response to the expert feedback. The questionnaire was then revised a second time, in response to the internal consistency results of the first pilot, as well as the response from the students’ evaluations during the interview. A second pilot test was conducted and the Cronbach alpha was measured a second time.

### Translation and back-translation

The questionnaire was developed in British English, but needed to be translated to Brazilian Portuguese for the pilot studies because they were conducted in Brazil. One of the authors, who is a native Brazilian and also fluent in English, translated the questionnaire to Brazilian Portuguese. An independent researcher, who is a geneticist and also bilingual in Brazilian Portuguese and British English, translated the Portuguese version back to English, in a process called “back translation” (the resulting questionnaire is herein called the ‘back-translation’). This was done in order to validate the translation. The authors then compared the back-translation with the original English version to see how similar they were. The two versions were overall very similar, but the comparison revealed some ambiguity in the meaning of a few of the items. The ambiguities were discussed amongst the translators and authors, and necessary changes were made in both the original English version and the Portuguese version. For example, the word “diet” in the original English version had to be changed to “eating habits”, otherwise the direct translation of “diet” in Portuguese would be confused with “being on a diet”.

### Participants and pilot studies

The first pilot study of the questionnaire was conducted in November 2014 in order to validate the questionnaire after the first revision. The questionnaire was administered to 207 first-year students enrolled in an Interdisciplinary Bachelor Program at a Brazilian Federal (public) university in Northeast Brazil. The majority (33%) of the students were studying humanities as their main field of study, followed by arts (25%), health sciences (22%), and science & technology (18%).

We chose first-year university students because they are representative of young adult Brazilians who have completed high school. It was important that they had obtained high school education (typically at an age of 16–18 years), so that they would have a level of knowledge about genetics and genomics that would allow for a more powerful study. Moreover, as we are interested in investigating attitudes toward genetic- or genomic-based technologies, Brazilian citizens who completed high school are more likely to be in the population with an income that could allow them to benefit from these technologies, given unequal access to them, and thus they are more likely to have formed some attitude towards them. Alternatively, we could have chosen students at the end of high school, but we considered that first-year university students would have almost the same amount of knowledge, and it would be easier to sample them in a single university.

Our sample is representative of an “educated youth” in Brazil, and is by no means representative of all Brazilians. There may be cultural variations within a country, and the questionnaire is most likely less applicable to groups of society who are less educated, such as those who did not finish high school. However, by using freshmen students we have a population of citizens that have taken high school, which is a large proportion of young adults. In Brazil 35.9% of the population 25 years of age or more have completed high school education, according to 2010 census data, IBGE 2010) [80]. The questionnaire will also be applicable in Western societies where freshmen university students represent the vast majority of youths.

The questionnaires were administered to the students during regular classes, at the end of their first term in the university.

They were asked to fill in the questionnaire whilst two of the authors of this paper were present. The questionnaire took on average 20 to 25 minutes to complete (with a few students being as quick as 13 minutes, and some taking up to 40 minutes).

A second pilot study was conducted after the second revision. We administered the second pilot to a new set of first-year students from the Interdisciplinary Bachelor Program at the same university, during February 2015. 78 students answered the questionnaire in the second pilot study. Of these, the majority were studying science and technology (33%) as their main field, followed by humanities (30%), health (17%), arts (15%) or other (4%). They were recruited during their enrollment, whilst they were waiting in line. We invited them to fill in the questionnaire in a separate classroom.

All participants gave written informed consent before answering the questionnaire and being interviewed (the informed consent form was translated from Portuguese into English and is provided in the S1 Text. All participants signed two forms, being one of them kept by the participant, the other by the researchers. This consent procedure was approved by the Committee for Ethics in Research from Federal University of Bahia Nursing School). Their names are anonymous on the questionnaires and in all data files (their names are replaced by numbers).

### Statistical analyses

The completed questionnaires were analyzed for internal consistency (reliability) using Cronbach alpha with the statistical packages SPSS (version 22) and R (with the *Psych* package) (all calculations were run in both, to ensure accuracy). All the questionnaires with more than 8 missing answers (10% of the total) were eliminated from the sample. Since the calculation of the Cronbach alpha does not support missing answers, we used modal value imputation method to replace the missing data [81].

For the analyses, we used Code Books that are shown in S4 Table and S2 Text. Raw data are shown in S5 and S6 Tables.

## Results

### Content validity

#### Evaluation by experts: First revision of the PUGGS questionnaire

Following the advice from the experts, we firstly simplified the core ideas by removing references to specific diseases or examples, to represent principles rather than detailed knowledge. Some of the core ideas were removed because they were considered too difficult by many of the experts, in the sense that they related to specific scientific knowledge that would either not be relevant from a public understanding perspective, or would be beyond the grasp of lay people or even basic education students. We also added a core idea about the levels of organization involved in the gene-phenotype relationship, as suggested by several of the experts. We kept two items per core idea for the section on genetic determinism, and in the section on modern genetics and genomics we increased to three items per core idea, to allow for future analyses of associations between the subsections of that section.

Some very minor adjustments were made to the background information (section 1) of the questionnaire. For section two of the questionnaire (table of traits), our initial version had a list of traits and disorders where respondents were asked to mark, on a scale from 0 to 10, the relative importance of environment and genes for each trait (from 0: Environment is most important to 10: Genes are most important). Several of the experts pointed out problems with this scale, mostly because it reflected a heritability scale (0 to 100% heritability). They advised us to reduce the number of points on the scale, and to use simpler categories. We therefore reduced the scale from a 10-point to a 5-point Likert format (from 1: *Only environmental differences contribute to the trait*, to 5: *Only genetic differences contribute to the trait*).

For section three (gene-environment interaction), the general advice from the experts was to make the items shorter, simpler, and less specific, so that detailed knowledge about specific traits or diseases would not be required from the respondents. For instance, the original item 4 read as follows: “A gene can be associated with several different traits—for example, the breast cancer gene mutation (BRCA2 gene) is associated with prostate cancer and melanoma”. In the revised version this item became: “A single gene can influence several different health problems”. On the basis of the experts’ responses, a majority of the items were modified, some were removed altogether, and some new items were added, improving content validity.

The items in section 4 (Knowledge about modern genetics and genomics) and section 5 (Attitudes) received fewer critical comments by the experts, and were generally considered satisfactory.

#### Evaluation through students’ interviews during the pilot testing

Of the nineteen students that were interviewed about their comprehension of the questionnaire items, ten said they did not understand the items on epigenetics, because they did not know what epigenetics is. We also asked them if there were any words they did not understand, and ten of the students said they had never heard of epigenetics, two said they had not heard of Tourette’s syndrome (one of the diseases in the table of traits) and one said he did not know what pharmacogenomics is. Consequently, we removed the item “Tourette’s syndrome” from the table of traits task, but we kept the items on epigenetics and pharmacogenomics, because even though students had not heard of them, they understood what these items were asking. Moreover, their knowledge on epigenetics and attitudes towards pharmacogenomics are part of the data the questionnaire intends to gather. Apart from these remarks, all the other questions were understood. Seven of the students did not report any problems at all.

### Internal consistency

#### After the first revision of the PUGGS questionnaire

The results of the internal consistency of the PUGGS questionnaire after the first revision are shown in Table 1. We tested the effect that removing certain items would have on the Cronbach alpha for most of the sections, and used these predictions as a guide to help us decide which items to revise or remove in the second pilot. The Cronbach alpha for the items on gene-environment interaction in (Section three) was poor (0.52), indicating that the items in this section were not all measuring what they were supposed to measure. Further revision on this section was therefore necessary, and, due to the number of changes, we recalculated the Cronbach alpha for this section based on the results from the second pilot. The reliability measures for the other sections were considered acceptable, although removing certain items improved the alpha slightly (see Table 1).

**Table 1:** Cronbach alpha results of the first pilot test and decision-making about revision of the PUGGS questionnaire.

Items	Cronbach alpha of pilot study	Cronbach alpha after removing some items	Decision for the final version
Belief in genetic determinism (Table of traits, Section 2)	0.67	No specific improvement	Three items removed due to many students answering “don’t know”. Minor changes in formulation
Gene-environment interaction (Section 3)	0.52	0.67 if four items are removed.	The four items have been removed and several items modified to improve clarity. Due to deep changes Cronbach alpha has been retested in the second pilot; result: 0.67
Knowledge about modern genetics and genomics (Section 4)	0.69	0.7 if one item was removed	The item has been removed, as well as another item, due to potential inaccuracy of the content.
Attitudes (whole of section 5)	0.85		
Gene therapy	0.62	0.8 if one item was removed	The item was modified to improve clarity
Genetic testing	0.54	0.72 if two items were removed	The two items were modified to improve clarity
Prenatal genetic testing	0.78	No specific improvement	Minor changes in the formulation in one item in order to remove double negative
Personalized medicine & pharmacogenomics	0.74	No specific improvement	No changes

#### Second revision of the PUGGS questionnaire

On the basis of the Cronbach alpha results, as well as the students’ feedback in the interviews, the questionnaire was revised a second time. We also explicitly attended to items with a high frequency of “don’t know” responses, and assessed whether the wording of these items were confusing, thus making them difficult to comprehend, or if the content was simply too difficult. We also used the student interviews to address these issues, since the students had the possibility to address all questions and their difficulties. We did not explicitly consider the number of correct and incorrect answers to each item during the revision process, because we felt that the students’ feedback in the interviews and the number of “don’t’ know” responses addressed the issue of item difficulty more adequately. However, in most cases the number of respondents answering “correct” on an item was lower if the item had a high amount of “don’t know” responses. Examples of this are given below.

For the table of traits (section 2) we removed the items “coronary heart disease”, “haemophilia”, and “Tourette´s syndrome” because over 15% of the respondents answered “Don’t know” on these. Moreover, in the interviews two students said they had never heard of Tourette’s syndrome.

The original thirteen items on gene-environment interaction (Section 3) underwent the most extensive revision, since they had the lowest estimate of reliability (alpha 0.52). In order to avoid unnecessary confusion, we removed double negatives (items with a negative statement that probed for a “disagree” answer in order to remain positive). For instance, the item “Traits and diseases caused by a single gene are not very common” was modified to “Most human traits and diseases are caused by a single gene”. This item also had 26% “Don’t know” answers and only 19.3% correct answers in the first pilot, indicating that it was difficult to understand—but this increased to 78.8% correct answers in the second pilot. We also removed potentially difficult examples in items, *e*.*g*., diabetes, Alzheimer’s disease, personality, intelligence, either by substituting the item with a more general statement, or by substituting a specific example with a more common trait. For instance, an item on Alzheimer’s disease had the highest amount of “Don’t know” answers (36%), and only 37.2% correct answers, so we changed it from “Alzheimer’s disease is influenced by one gene only” to “A person’s height is influenced by one gene only”. The number of correct answers in the second pilot increased to 56%.

We also tried to improve the readability and clarity of the items in Section three by making them simpler and shorter. We deleted altogether four items that were either too complicated, or potentially misleading. This included an item on levels of biological organization, because it was too long and complicated (with 18.8% “Don’t know” answers); an item on diabetes, because it was too specific and also redundant; and two items on mutations, because they were too difficult for the intended respondents (we also removed the corresponding core idea). For the remaining items the amount of “Don’t know” answers was fairly low (ranging from 0.5% to 9.7%).

The Cronbach alpha in Section 4 (Modern Genetics and Genomics) was good (0.69) and it was not necessary to make many changes in the corresponding items. However, we removed two items due to potential inaccuracy: for instance, the item “Humans have about the same amount of genes as a fruit fly”. We made some minor changes in the formulation of some of the items to improve clarity, for instance by removing double negatives (e.g. “Epigenetic changes are not influenced by environmental factors” was modified to “Epigenetic changes are influenced by environmental factors”). Respondents frequently answered “Don’t know” to many of the items in this section. In particular, between 43% and 58% respondents answered “Don’t know” on the items on epigenetics. Despite this, we decided to keep these items since they represent newly established concepts in science and society, and answering “Don’t know” to newer concepts can give an indication as to whether people have heard about them or not. This is important in the context of this instrument since an understanding of epigenetics (involving an idea on how environmental factors can influence gene activity) could be a possible factor inhibiting genetic deterministic beliefs.

In the Attitudes section, very few students answered “Don’t know” (ranging from 0% to 5.8%), indicating that they understood what was being asked in these items. This was also confirmed in the students’ interviews. Also, the overall Cronbach alpha for the Attitudes section was very good (0.85). We therefore did not change much in this section, except for some of the wording—in particular, we modified double negatives, since they are potentially confusing. For example, in the subsection of gene therapy the item “I would be worried about gene therapy being used to modify or enhance physical attributes such as athletic performance” was modified to “I think gene therapy should be used to modify or enhance physical attributes such as athletic performance”. There were also two items in the subsection on genetic testing that had double negatives and this sub-section had the lowest Cronbach alpha score (0.54) of the four attitude topics. These items were subsequently modified so that they would be easier to comprehend. Although removing these items would have had a positive effect on the Cronbach alpha for each of these subsections, we opted to modify instead of removing them because we think that the items cover important issues within genetic testing and gene therapy.

We also changed some of the answer options: Since the questions on gene-environment interaction (Section 3) and modern genetics and genomics (section 4) are predominantly knowledge questions, we changed the modalities for these from a Likert to a True/False response format: In the first pilot, we used a Likert scale for all items, with the options “strongly disagree/disagree/agree/strongly agree/don’t know”. This was changed to a True/False (and “Don’t know”) format for sections 3 and 4 in the second pilot. We decided to include the “Don’t know” option because it gives valuable information about the topics and items students know less about. We removed it from the Attitudes section (Section 4), since very few students marked this option. We also decided to remove the “Don’t know” option from the table of traits task (Section 2) since this task is capturing belief in determinism, rather than pure knowledge, and therefore assume that most students can answer this task regardless of how much knowledge they have.

#### After the second revision

After the questionnaire had been revised a second time, another pilot test among a new sample of students (n = 78) was conducted and the Cronbach alpha was measured a second time for the parts of the questionnaire that had undergone substantial revision (Section 3 on gene-environment interaction). The new alpha score for the items on gene-environment interaction was 0.67, which was acceptable (Table 1). This indicates that the second revision had significantly improved the reliability of these items and we decided that no more revision was necessary. The final version of the PUGGS questionnaire contained 45 items and 11 core ideas.

## Discussion

We have developed and validated a questionnaire instrument (the PUGGS questionnaire) that can be used to test young adults’ understandings and attitudes towards modern genetics and genomics, levels of belief in genetic determinism, as well as to investigate correlations between them. To our knowledge, no such instrument currently exists, and therefore the PUGGS questionnaire fills an important gap in research on genetics education and public understanding about genetics.

The instrument underwent two revisions in order to reach an acceptable measure of validity and reliability. Prior to the first revision a group of seventeen researchers from a variety of fields including genetics, genetics education, and history, philosophy, and sociology of science reviewed the proposed items and provided suggestions for improvements. This resulted in an initial revision that improved the content validity.

The instrument was then piloted to 207 undergraduates in a Brazilian Federal university and the internal reliability was measured, revealing weak consistency among the items measuring gene-environment interaction (Section 3). The questionnaire therefore underwent a second revision and second pilot test (with 78 freshmen students from the same university), paying particular attention to those items measuring gene-environment interaction, but also generally improving the readability and clarity of the entire instrument. This improved the internal reliability of Section 3 to an acceptable level (Cronbach alpha of 0.67). The other sections already had a good measure of reliability in the first pilot (0.67 for section 2 on Belief in genetic determinism, 0.69 for Section 4 on knowledge about modern genetics and genomics, and 0.85 for Section 5, on attitude towards applications of modern genetics and genomic-based technologies, see Table 1), and thus did not need much revision. Overall, the PUGGS questionnaire has good reliability and validity, although there are some differences between the different sections of the instrument, as discussed below.

### Genetic Determinism

In the section measuring beliefs in genetic determinism (Section 2), students were presented with a list of common traits and asked to indicate the extent they thought genetic and environmental differences contributed to each trait, answering on a Likert response format. Our scale includes five points (1: only environmental, 2: mainly environmental, 3: both to the same extent, 4: mainly genetic, 5: only genetic). A previous study by Molster et al. [48] had only three categories (All genes—Mix—All environment), in which the middle category naturally covers many different types of traits; some are more genetic (*e*.*g*., heart disease) and some are more environmental (*e*.*g*., depression). We opted for two extra categories on either side of the middle category, in order to accommodate for this variety and to allow for more interesting results (otherwise almost all the traits would fall into the middle category). One could argue that, in reality, no traits are either “only environmental” or “only genetic”, and that these categories are therefore misleading, but we had to simplify the categories somewhat in order to measure high or low scores in beliefs in determinism.

One way of using the table of traits in future analyses might be to present a chart of all the respondents’ answers, in order to visualize the responses. A possible way of analyzing the answers could be to pick out the traits that are more obviously environmental or more obviously genetic, and see how the respondents’ answers compare to a scientific interpretation or consensus. For instance, if a respondent answers “largely genetic” to “interest in fashion” (which is mostly environmental) this would indicate a strong deterministic view.

The set of nine questions that followed the table of traits were designed to measure the respondents’ knowledge about gene-environment interaction, as a way of obtaining additional information related to putative commitments to genetic determinism. For instance, more knowledge about the interaction between genetic and environmental factors in the development of traits would indicate less determinism. This section yielded an acceptable Cronbach alpha (0.67) after the second pilot (Table 1). Compared to other determinism scales, which tend to have Cronbach alphas in the range of 0.7–0.8 [61, 77], the Cronbach alpha for these nine items in the PUGGS questionnaire is somewhat lower. This may be because our items more strongly emphasize the scientific grounding in genetics than in many other scales. Indeed, it is common for other scales to include items reflecting determinism on a more social or political level, with examples from everyday life such as “When people know their genetic make-up they will take less responsibilities” or “People’s knowledge of their genetic make-up will decrease their self-confidence” [61]. Perhaps these types of “everyday” scenarios are easier for people to relate to than a context-independent statement such as “Most human traits and diseases are caused by a single gene” (from the PUGGS questionnaire), which does not relate directly to people’s experiences. However, we think it is a good idea to measure knowledge related to gene-environment interaction (with bearings upon the subjects’ commitments to genetic determinism) by using more context-independent statements, because when one uses an item involving social or political issues, it may become unclear what is really influencing the measurement, whether it is deterministic thinking or some other belief in the social or political issues at stake.

Another possible explanation for why genetic determinism is difficult to measure, is that perceptions of the relationship between genes and traits are not only rooted in people’s knowledge, but also in their values and social practices [16], making it harder to measure it in a coherent way. For instance, Dambrun and colleagues [35] has shown that university experiences in the social sciences lead students to the perception that nature plays a less important role than nurture in shaping human behavior and personality. Social science education therefore seems to make people less prone to genetic deterministic thinking and more inclined towards environmental deterministic thinking. Furthermore, Keller [34] has argued that “geneticism” (genetic determinism) is strongly related to a variety of ideologies including patriotism, nationalism, the protestant ethic, modern sexism, racial stereotyping, racial prejudice, and anti-egalitarianism. It therefore seems that genetic determinism is a result of the interaction between many different types of knowledge and values, which explains why it is in itself a difficult construct to measure. This may also explain difficulties in measuring knowledge about gene-environment interactions, with which belief in genetic determinism interferes.

In sum, determinism scales tend to be developed in the context of particular topics, and as part of larger studies, and are probably not very reliable for use on their own. Therefore, we advise others to use items on determinism in the PUGGS questionnaire in combination with the other sections of the instrument.

### Knowledge about modern genetics and genomics

The overall Cronbach alpha for the section on modern genetics and genomics was 0.7 (Table 1), indicating that the reliability is good. There were particularly high amounts of “Don’t know” answers in the questions on epigenetics, corroborating a recent British study that found that only two of 154 secondary school students aged 14 to 16 had heard of the term epigenetics [82]. We decided to keep the items on epigenetics, despite the apparent lack of knowledge about it, because these items will allow for future analyses to assess the extent with which students are keeping up with modern developments in genetics and genomics.

Moreover, knowledge of concepts involved in epigenetics is becoming increasingly important for understanding modern medicine, such as personalized medicine, and direct-to-consumer genetic testing. The genetics education community calls for a greater emphasis on the teaching of genomics in the high school science curriculum, with less focus on transmission genetics and Mendelian inheritance [44, 52]. Despite this, evaluations of the U.S. state standards in genetics teaching show that, from a list of genetic literacy benchmarks, only evolution and the nature of genetic material are adequately covered in the U.S. high school genetics curriculum, while topics such as gene expression and regulation, and genetic variation, are not properly addressed [71]. In Brazil, themes related to contemporary genetics are not often present in textbooks, which play a key role in teachers’ practice and students’ learning [18, 83, 84]. This may explain, therefore, why many of the students in the first pilot test answered “Don’t know” in the section of the questionnaire on modern genetics and genomics. Future analyses using this scale may reveal results that will be of use for curriculum development and design.

### Attitudes towards applications of modern genetics and genomics

The overall Cronbach alpha for the whole section on attitudes was 0.85, indicating very good reliability. This means that we can assume that the items in the attitudes section are measuring what they are supposed to measure.

The answers in the attitudes section indicated that the students were well aware of the various applications of modern genetics and genomics, since very few answered “Don’t know” to the attitude questions (in the first pilot). Therefore, the students were able to give their opinions concerning the topics in question.

Two of the topics in the attitudes section covered personalized medicine and genetic testing, which are becoming increasingly common in public health. In 2010 roughly 10% of the drugs approved by the U.S. Food and Drug Administration had labels with pharmacogenomics information, and genetic testing is common for certain conditions such as breast cancer [85]. Genetic testing, gene therapy and new cancer drugs tailored to patients’ genes are also commonly mentioned in the media and, therefore, it is not surprising that students seem acquainted with these topics. It will be interesting in further studies to investigate how public attitudes towards genomic technologies correlate with knowledge of modern genetics and genomics and commitment to deterministic thinking.

### Applications of the PUGGS questionnaire

The PUGGS questionnaire was developed and validated among college freshmen students in Brazil, and we consider it generalizable to other populations of young adults with at least a high school education in most Western societies. We consider this the first validation step in an ongoing work, and additional studies are needed to validate it for other population segments/groups. We therefore encourage others to apply the questionnaire in different contexts to secure the generalizability. For example, the current research group are already planning to test out the questionnaire among physicians, to see how their knowledge compares to college students.

In future studies, we also suggest that the PUGGS questionnaire can be used to analyze the relationship between beliefs in genetic determinism, knowledge about genetics and genomics, and attitudes toward genetic and genomic-based technologies. For example, one could compare respondents with high and low scores in knowledge of modern genetics and genomics and see whether they score differently in beliefs about genetic determinism. It may also be possible to inquire into how genomic knowledge or belief in genetic determinism is related to attitudes towards genetic and genomic-based technologies. Evidently, other associations can also be explored. A practical benefit of such research could be in science education, where educators could use the questionnaire to map students’ knowledge, attitudes and belief in genetic determinism, and tailor the classroom instruction accordingly. It may also be important for teachers to find out how knowledge and attitudes about modern genetics shape each other, so that potential obstacles to learning may be addressed.

The instrument may be applied to diverse groups of people, for example, for measuring knowledge and attitudes towards modern genetics and genomics among health personnel, genetic counselors, biology or medical university students, educators, health communicators, or journalists. Multinational comparative studies will also be possible, especially in the context of genetics education and genetic literacy, where analyses in some regions or countries may reveal areas of knowledge or attitudes that demand attention.

The full version of the PUGGS questionnaire is available in the S1 Table.

## Supporting Information

S1 Table The PUGGS questionnaire. (DOCX)

S2 Table The core ideas. (DOCX)

S3 Table Initial PUGGS questionnaire, used in the first pilot study. (DOCX)

S4 Table Code Book used in the first pilot study of the PUGGS questionnaire. (DOCX)

S5 Table Raw data from the first pilot. (XLSX)

S6 Table Raw data from the second pilot. (XLSX)

S1 Text Informed consent form. (DOCX)

S2 Text Code Book used in the second pilot study of the PUGGS questionnaire. (DOCX)
