# Supplementary material for: Young Adults’ Belief in Genetic Determinism, and Knowledge and Attitudes towards Modern Genetics and Genomics: The PUGGS Questionnaire
Source: PLoS One. 2017 Jan 23;12(1):e0169808. doi: 10.1371/journal.pone.0169808 (PMC5256916; doi:10.1371/journal.pone.0169808)
Supplement: S1 Table — (DOCX) [file pone.0169808.s001.docx]

Supporting Information 1

Public Understanding and Attitudes towards Genetics and Genomics (PUGGS) questionnaire

# Section 1: Background information

Please mark with an X the appropriate answer:

1. What is your age?

- 15 or younger
- 16-18
- 19-21
- 22 or older

1. What is your gender?

- Male
- Female
- Other (transgender, transexual, etc.)

1. What is your main field of study at the university?

- Science and technology
- Humanities
- Health
- Arts
- Other. Please specify: __________________________________________________

1. In what year did you start University? _____________________
2. In general, to what extent are your opinions and decisions influenced by religion?

- Greatly influenced
- Somewhat influenced
- Not influenced

1. Have you or anyone close to you ever had any experience with genetic issues? For example, having a heritable disease in the family, or taking a genetic test?

- Yes
- No

If yes, please add further information in the box below, specifying what kind of experience you or anyone close to you have had with genetic issues.

# Section 2: Belief in genetic determinism

| *For each trait mark with an “X” in ONLY ONE of the columns from 1 to 5.* | Only environ-mental differences contribute to the trait | Mainly environ-mental differences contribute to the trait | Both genetic and environ-mental differences contribute to the same extent to the trait | Mainly genetic differences contribute to the trait | Only genetic differences contribute to the trait |
| --- | --- | --- | --- | --- | --- |
|  | **1** | **2** | **3** | **4** | **5** |
| *Example: Eye colour* |  |  |  |  | ***X*** |
| Height |  |  |  |  |  |
| Bipolar disorder |  |  |  |  |  |
| Diabetes |  |  |  |  |  |
| Colour blindness |  |  |  |  |  |
| Schizophrenia |  |  |  |  |  |
| Alcoholism |  |  |  |  |  |
| Breast cancer |  |  |  |  |  |
| Interest in fashion |  |  |  |  |  |
| Addiction to gambling |  |  |  |  |  |
| Political beliefs |  |  |  |  |  |
| Intelligence in adults |  |  |  |  |  |
| Severe depression |  |  |  |  |  |
| Attention Deficit Hyperactivity Disorder (ADHD) |  |  |  |  |  |
| Asthma |  |  |  |  |  |
| Violent behaviour |  |  |  |  |  |
| Religious beliefs |  |  |  |  |  |
| Blood group (ABO) |  |  |  |  |  |

People vary in traits (physical features, behaviours, diseases and disorders), such as those shown in the table below. Genetic differences and environmental differences contribute to this variation. Environmental differences can for example be differences in culture, upbringing, lifestyle, eating habits, or exposure to pollution. In the table below please indicate to what extent you think genetic and environmental differences contribute to these traits.

# Section 3: Knowledge about gene-environment interaction

| Q. | *Please read each statement below and tick one box (True, False or Don´t know).*  ***N.B. Please only choose “don’t know” if you do not understand the statement.*** | True | False | Don’t know |
| --- | --- | --- | --- | --- |
| 1 | A gene codes directly for a trait or disease. |  |  |  |
| 2 | Most human traits and diseases are caused by a single gene. |  |  |  |
| 3 | A single gene can influence several different traits or diseases. |  |  |  |
| 4 | A person´s height is influenced by one gene only. |  |  |  |
| 5 | Most traits and diseases are influenced by many different genes. |  |  |  |
| 6 | Most traits and diseases are caused by environmental factors only (such as diet and lifestyle). |  |  |  |
| 7 | A gene can only influence a single trait or disease. |  |  |  |
| 8 | Most traits and diseases are caused by both genes and environmental factors. |  |  |  |
| 9 | A person´s height is influenced by many different genes. |  |  |  |

## Section 4: Knowledge about modern genetics and genomics

| Q. | *Please read each statement below and tick one box (True, False or Don´t know).*  ***N.B. Please only chose “don’t know” if you do not understand the statement*** | True | False | Don’t know |
| --- | --- | --- | --- | --- |
| 10 | The genome consists only of the genes in an organism that code for the production of proteins. |  |  |  |
| 11 | Cells, tissues and organs differ because they have different sets of genes that are activated (“turned on”) and deactivated (“turned off”). |  |  |  |
| 12 | Environmental factors, such as cigarette smoke, can affect gene activity. |  |  |  |
| 13 | The human genome contains more genes than the genome of any other living being. |  |  |  |
| 14 | Every cell of the body contains the whole genome. |  |  |  |
| 15 | When someone says something is “epigenetic” it means that you can inherit changes in gene activity without inheriting changes in the genes. |  |  |  |
| 16 | Only a small proportion of the human genome consists of genes that code for proteins. |  |  |  |
| 17 | Epigenetic changes are influenced by environmental factors. |  |  |  |
| 18 | When someone talks of an epigenetic change he or she is referring to a large change in the DNA sequence. |  |  |  |
| 19 | Epigenetic changes are caused by mutations. |  |  |  |
| 20 | Most of the human genome consists of genes that code for proteins. |  |  |  |
| 21 | The human genome has fewer genes than some less complex organisms such as tomato plants and rice. |  |  |  |
| 22 | When someone says something is “epigenetic”, it means that environmental factors can change part of the DNA sequence |  |  |  |
| 23 | Genes can be activated or deactivated by other genes. |  |  |  |
| 24 | Only eye cells have genetic information for eye colour. |  |  |  |
| 25 | If a cell lacks a certain substance, such as a vitamin, a gene can be deactivated. |  |  |  |

# Section 5: Attitudes towards applications of modern genetics and genomics

## Gene therapy

Gene therapy involves the replacement of a faulty gene in an affected tissue in the body by a normally-functioning gene. Research is still at the clinical trial stage, but recent advances are offering new hope for future treatment in humans.

| Q. | *On a scale from strongly disagree to strongly agree, to what extent do you agree with the following statements?*  ***N.B. Please only chose “don’t know” if you do not understand the statement.*** | Strongly disagree | Disagree | Agree | Strongly agree |
| --- | --- | --- | --- | --- | --- |
| 26 | I am skeptical toward gene therapy because I am scared by the thought of interfering with our genes |  |  |  |  |
| 27 | If I had a serious genetic disorder I would consider undergoing gene therapy to try to cure it. |  |  |  |  |
| 28 | I would be glad if gene therapy was available for people with serious genetic disorders. |  |  |  |  |
| 29 | I think gene therapy should be used to modify or enhance physical attributes such as athletic performance. |  |  |  |  |
| 30 | I am generally positive towards gene therapy and think the government should invest more money into its development. |  |  |  |  |

## Genetic testing

A genetic test examines your DNA, and can reveal changes or variations in your genes that may be associated with an illness or a disorder. A genetic test can be arranged by your doctor or health clinic, or in some countries you can order a genetic test yourself on the internet (called “direct-to-consumer” testing).

| Q. | *On a scale from strongly disagree to strongly agree, to what extent do you agree with the following statements?*  ***N.B. Please only chose “don’t know” if you do not understand the statement*** | Strongly disagree | Disagree | Agree | Strongly agree |
| --- | --- | --- | --- | --- | --- |
| 31 | At some point in my life, I might consider having a genetic test to find out my risk of developing various genetic diseases. |  |  |  |  |
| 32 | I am glad that genetic tests can be ordered on the internet. |  |  |  |  |
| 33 | I am glad that genetic tests are available so that people with a family history of serious genetic disease can find out if they are at risk. |  |  |  |  |
| 34 | The availability of genetic tests for insurance companies and future employers is problematic. |  |  |  |  |
| 35 | I am generally positive towards genetic testing and think the government should invest more money into its development. |  |  |  |  |

## Prenatal genetic testing

Prenatal genetic diagnosis is used to detect changes or variations in a fetus’s genes or chromosomes before birth, to see if it has any chromosomal or genetic defects. This can help parents know more about the future possibilities in the life of their children.

| Q. | *On a scale from strongly disagree to strongly agree, to what extent do you agree with the following statements?*  ***N.B. Please only chose “don’t know” if you do not understand the statement.*** | Strongly disagree | Disagree | Agree | Strongly agree |
| --- | --- | --- | --- | --- | --- |
| 36 | If I had a family history of a serious genetic disease, I would definitely want to use prenatal genetic diagnosis. |  |  |  |  |
| 37 | I do not think prenatal diagnosis should be made available for detecting conditions such as asthma and Attention Deficit Hyperactivity Disorder (ADHD). |  |  |  |  |
| 38 | The government should make prenatal genetic testing available to all individuals who want it. |  |  |  |  |
| 39 | Prenatal genetic testing should only be allowed for severe genetic diseases. |  |  |  |  |
| 40 | I am generally positive towards prenatal genetic diagnosis and think the government should invest more money into its development. |  |  |  |  |

## Personalised medicine and pharmacogenomics

Genetic testing is the first step in personalized medicine and pharmacogenomics. Personalised medicine uses knowledge of a person’s genes to predict his or her risk for developing a particular disease and to influence decisions about lifestyle in order to help prevent or manage a disease. Pharmacogenomics uses genetic information to find the best medicine or treatment for a disease, for example when doctors predict how an individual’s genes will respond to certain cancer drugs. In some cases doctors analyze particular genes associated with the cancer, and in other cases they may analyze the entire genome of the individual.

| Q. | *On a scale from strongly disagree to strongly agree, to what extent do you agree with the following statements?*  ***N.B. Please only chose “don’t know” if you do not understand the statement*** | Strongly disagree | Disagree | Agree | Strongly agree |
| --- | --- | --- | --- | --- | --- |
| 41 | If I were diagnosed with cancer, I would consider having my genes analysed in order to help chose a cancer treatment with the fewest side effects. |  |  |  |  |
| 42 | If I had a family history of diabetes I would consider having my genes analysed in order to help me make lifestyle choices and decisions about interventions that may prevent diabetes from developing. |  |  |  |  |
| 43 | I would not be willing to get my whole genome analysed, because I worry about issues of confidentiality. |  |  |  |  |
| 44 | I am sceptical toward pharmacogenomics because of the possibility of getting information about my genes that is unrelated to the treatment. |  |  |  |  |
| 45 | I am generally positive towards personalized medicine and pharmacogenomics and think the government should invest more money into its development. |  |  |  |  |
