# Supplementary material for: Young Adults’ Belief in Genetic Determinism, and Knowledge and Attitudes towards Modern Genetics and Genomics: The PUGGS Questionnaire
Source: PLoS One. 2017 Jan 23;12(1):e0169808. doi: 10.1371/journal.pone.0169808 (PMC5256916; doi:10.1371/journal.pone.0169808)
Supplement: S2 Table — (DOCX) [file pone.0169808.s002.docx]

Supporting Information 2

# S2 Core ideas. Core ideas with corresponding items and sources

## Section 3: Knowledge about gene-environment interaction

| **Core idea** | **Description (what the core idea seek to test):** | **Corresponding items (and item number)** | **Sources** |
| --- | --- | --- | --- |
| **A.** Most traits and diseases are polygenic (caused by many different genes); far fewer traits and diseases are monogenic (caused by changes in a single gene). | Whether respondents are aware that *most* traits are polygenic – determined by many genes. | (2) Most human traits and diseases are caused by a single gene. (False)  (5) Most traits and diseases are influenced by many different genes. (True) | (Bowling et al., 2008; Hott et al., 2002; M. U. Smith, 2014) |
| **B.** One gene can influence several different traits and diseases (pleiotropy). | Awareness that the causal relationship between genes and traits is more complex than a one-to-one relationship. | (3) A single gene can influence several different traits or diseases. (True)  (7) A gene can only influence a single trait or disease.(False) | (Aivelo, 2015; Dougherty, 2009)  + item developed by authors |
| **C.** One trait or disease can be influenced by several genes. | Awareness that a trait may be polygenic – determined by many genes. This is similar to core idea A, but does not specify the quantity/proportion of traits like this. | (4) A person´s height is influenced by one gene only. (False)  (9) A person’s height it influenced by many different genes. (True) | (Aivelo, 2015; Bowling et al., 2008; Dougherty et al., 2011; M. U. Smith, 2014) |

| **Core idea** | **Description (what the core idea seek to test):** | **Corresponding items (and item number)** | **Sources** |
| --- | --- | --- | --- |
| **D.** Most traits and diseases are caused by the interaction between many genes and environmental factors. | Awareness of the multifactorial process; that environmental and epigenetic factors also play a role in the gene-trait relationship. | (6) Most traits and diseases are caused by environmental factors only (such as diet and lifestyle). (False)  (8) Most traits and diseases are caused by both genes and environmental factors. (True) | (Bowling et al., 2008; Dougherty et al., 2011; Hott et al., 2002; M. U. Smith, 2014) |
| **E.** A gene’s influence on a trait starts with proteins, but the resulting trait can be described on different levels of biological organization; the trait is the outcome of a multifactorial developmental process. | Similar to core idea D, but adds the dimension that there are several “steps” between genes and traits, reflected in a developmental process. | (1) A gene codes directly for a trait or disease. (False) | (Bowling et al., 2008; Duncan & Reiser, 2007; Sarkar, 2011; Shea, Duncan & Stephenson, 2015) |

## Section (4): Knowledge about modern genetics and genomics

| **Characteristics of the genome:** | | | |
| --- | --- | --- | --- |
| **Core idea** | **Description** | **Corresponding items** | **Sources** |
| **F.** A genome is an organism's complete set of DNA. It includes both the genes and the non-coding sequences of the DNA; only a small proportion of the human genome consists of protein-coding genes. | This is a definition of “a genome”. It tests whether respondents are aware that the genome has a lot of non-coding DNA sequences. | (10) The genome consists only of the genes in an organism that code for the production of proteins. (False)  (16) Only a small proportion of the human genome consists of genes that code for proteins. (True)  (20) Most of the human genome consists of genes that code for proteins. (False) | (Sarkar, 2011) |
| G. There is no correlation between the number of genes and the complexity of an organism; i.e. humans do not necessarily have more genes than other animals, such as plants, insects or birds. | This tests whether respondents are aware that the complexity of an organism is not just a result of the number of genes, but that there are other mechanisms involved (transcription & epigenetic factors etc., not mentioned here). | (13) The human genome contains more genes than the genome of any other living being. (False)  (21) The human genome has fewer genes than some less complex organisms such as tomato plants and rice. (True) | (Sarkar, 2011) |

| **Gene function and expression:** | | | |
| --- | --- | --- | --- |
| **Core idea** | **Description** | **Corresponding items** | **Sources** |
| H. Every cell of the body contains the same genome; what makes cells different is that different genes are expressed. | The main idea here is that all the body’s cells contain all our genes, but different genes are active in different cells at any given time. | (11) Cells, tissues and organs differ because they have different sets of genes that are activated (“turned on”) and deactivated (“turned off”). (True)  (14) Every cell of the body contains the whole genome. (True)  (24) Only eye cells have genetic information for eye colour. (False) | (Bowling et al., 2008; M. K. Smith, Wood & Knight, 2008) |
| I. Genes can be turned on and off by the influence of other genes, by substances present in the cell (such as signaling and transcription factors) or by environmental factors. | This idea is about what makes genes active or inactive. | (12) Environmental factors, such as cigarette smoke, can affect gene activity. (True)  (23) Genes can be activated or deactivated by other genes. (True)  (25) If a cell lacks a certain substance, such as a vitamin, a gene can be deactivated. (True) | (Bowling et al., 2008; Dougherty et al., 2011; Hott et al., 2002; M. U. Smith, 2014) |

| **Epigenetics:** | | | |
| --- | --- | --- | --- |
| **Core idea** | **Description** | **Corresponding items** | **Sources** |
| J. The term “epigenetics” encompasses mechanisms for heritable changes (in genetic activity and its subsequent effects) that do not involve alterations of the coding sequence of DNA. | This is a definition of the term “epigenetics”. It tests whether respondents understand the basic principle of epigenetics; that epigenetic changes do NOT involve changes in the DNA sequence. | (15) When someone says something is “epigenetic” it means that you can inherit changes in gene activity without inheriting changes in the genes. (True)  (18) When someone talks of an epigenetic change he or she is referring to a large change in the DNA sequence. (False)  (22) When someone says something is “epigenetic”, it means that environmental factors can change part of the DNA sequence. (False) | (Bird, 2007)  + items developed by the authors |
| K. Epigenetic changes can be triggered by environmental influences. | This idea is about what causes epigenetic effects. | (17) Epigenetic changes are influenced by environmental factors. (True)  (19) Epigenetic changes are caused by mutations. (False) | (Bird, 2007)  + items developed by the authors |

## References

Aivelo T, Uitto A. Genetic determinism in the Finnish upper secondary school biology textbooks. NorDiNa. 2015; 11(2):139-52.

Bird, A. Perceptions of epigenetics. Nature. 2007*;* 447(7143): 396-398. doi:10.1038/nature05913

Bowling BV, Acra EE, Wang L, Myers MF, Dean GE, Markle GC, et al. Development and evaluation of a genetics literacy assessment instrument for undergraduates. Genetics. 2008;178(1):15-22. doi: 10.1534/genetics.107.079533.

Dougherty MJ. Closing the gap: Inverting the Genetics curriculum to ensure an informed public. Am J Hum Genet. 2009; 85(1):6-12. doi: 10.1016/j.ajhg.2009.05.010.

Dougherty MJ, Pleasants C, Solow L, Wong A, Zhang H. A comprehensive analysis of High School Genetics Standards: Are states keeping pace with modern genetics? CBE Life Sci Educ. 2011;10(3):318-327. doi: 10.1187/cbe.10-09-0122.

Duncan RG, Reiser BJ. Reasoning across ontologically distinct levels: Students' understandings of molecular genetics. J Res Sci Teach. 2007; 44(7):938-59. doi: 10.1002/tea.20186.

Hott AM, Huether CA, McInerney JD, Christianson C, Fowler R, Bender H, et al. Genetics content in introductory Biology courses for non-science majors: Theory and practice. BioScience. 2002;52(11): 1024-1035. doi: 10.1641/0006-3568(2002)052[1024:gciibc]2.0.co;2

Sarkar S. Genomics, proteomics, and beyond. In: Sarkar S, Plutynski A, editors. A companion to the philosophy of biology. Malden, MA: Blackwell Publishing; 2011. pp. 58-74.

Shea N, Duncan R, Stephenson C. A tri-part model for genetics literacy: Exploring undergraduate student reasoning about authentic genetics dilemmas. Res Sci Ed. 2015; 45(4):485-507. doi: 10.1007/s11165-014-9433-y.

Smith MK, Wood WB, Knight JK. The Genetics Concept Assessment: A new concept inventory for gauging student understanding of genetics. CBE Life Sci Educ. 2008;7(4):422-430. doi: 10.1187/cbe.08-08-0045.

Smith MU. It's not your grandmother's genetics anymore! Am Biol Teach. 2014; 76(4): 224-229. doi: 10.1525/abt.2014.76.4.2.
