# Supplementary material for: Young Adults’ Belief in Genetic Determinism, and Knowledge and Attitudes towards Modern Genetics and Genomics: The PUGGS Questionnaire
Source: PLoS One. 2017 Jan 23;12(1):e0169808. doi: 10.1371/journal.pone.0169808 (PMC5256916; doi:10.1371/journal.pone.0169808)
Supplement: S3 Table — (DOCX) [file pone.0169808.s003.docx]

Supporting Information 3

Initial PUGGS questionnaire, used in the first pilot study

# Section 1: Background information

Please put an X in the relevant box:

1. What is your age group?

- 15 or younger
- 16-18
- 19-21
- 22 or older

1. What is your gender?

- Male
- Female
- Another option (transgender, transexual, etc.)

1. What is your main field of study at the university?

- Biology
- Other natural sciences
- Social sciences or humanities
- Arts
- Other. Please state: __________________________________________________

1. In general, to what extent are your opinions and decisions influenced by religion?

- Greatly influenced
- Somewhat influenced
- Not influenced at all

1. Have you or anyone close to you ever had any experience with genetic issues? For example, having a heritable disease in the family, or taking a genetic test?

- Yes
- No

If yes, please add further information in the box below, specifying what kind of experience you or anyone close to you had with genetic issues.

# Section 2: The influence of genes and environment on traits

## Section 2 Part 1

| ***For each trait put an “X” in ONLY ONE of the columns from 1 to 5.***  *Please only answer “don’t know” if you have not heard of the trait.* | Only environ-mental differences contribute to the trait | Largely environ-mental differences contribute to the trait | Both genetic and environ-mental differences contribute to the same extent to the trait | Largely genetic differences contribute to the trait | Only genetic differences contribute to the trait | Don’t know |
| --- | --- | --- | --- | --- | --- | --- |
|  | **1** | **2** | **3** | **4** | **5** |  |
| *Example: Eye colour* |  |  |  |  | ***X*** |  |
| Coronary heart disease |  |  |  |  |  |  |
| Height |  |  |  |  |  |  |
| Bipolar disorder |  |  |  |  |  |  |
| Diabetes |  |  |  |  |  |  |
| Colour blindness |  |  |  |  |  |  |
| Schizophrenia |  |  |  |  |  |  |
| Alcoholism |  |  |  |  |  |  |
| Breast cancer |  |  |  |  |  |  |
| Interest in fashion |  |  |  |  |  |  |
| Haemofilia blood disorder |  |  |  |  |  |  |
| Addictive gambling behaviour |  |  |  |  |  |  |
| Political beliefs |  |  |  |  |  |  |
| Intelligence in adults |  |  |  |  |  |  |
| Major depression |  |  |  |  |  |  |
| Tourette syndrome (tics disorder) |  |  |  |  |  |  |
| Attention Deficit Hyperactivity Disorder (ADHD) |  |  |  |  |  |  |
| Asthma |  |  |  |  |  |  |
| Violent behaviour |  |  |  |  |  |  |
| Religious beliefs |  |  |  |  |  |  |
| Blood group (ABO) |  |  |  |  |  |  |

People vary in traits (physical features, behaviours, diseases and disorders), such as those indicated in the table below. Genetic differences and environmental differences contribute to these variations. Environmental differences can for example be differences in culture, upbringing, lifestyle, diet, or exposure to pollution or radiation. In the table below please indicate to what extent you think genetic and environmental differences contribute to the traits mentioned.

## Section 2 Part 2

| Q. | ***On a scale from strongly disagree to strongly agree, to what extent do you agree with the following statements?***  *(NB: Please only chose “don’t know” if you do not understand the statement)* | Strongly disagree | Disagree | Agree | Strongly agree | Don’t know |
| --- | --- | --- | --- | --- | --- | --- |
| 1 | The majority of human traits and disorders are caused by a single gene. |  |  |  |  |  |
| 2 | Traits and disorders caused by a single gene are not very common. |  |  |  |  |  |
| 3 | A single gene can influence several health problems. |  |  |  |  |  |
| 4 | A gene can only influence only one type of health problem. |  |  |  |  |  |
| 5 | Alzheimer’s disease is influenced by one gene only. |  |  |  |  |  |
| 6 | Intelligence is influenced by many different genes. |  |  |  |  |  |
| 7 | Diet and exercise can play an important role in preventing and managing diabetes. |  |  |  |  |  |
| 8 | Personality is caused by genes only. |  |  |  |  |  |
| 9 | Most traits and disorders are caused by both genes and environmental factors. |  |  |  |  |  |
| 10 | A gene holds the information that codes directly for a trait such as height, eye colour or colour blindness. |  |  |  |  |  |
| 11 | A gene bears the information that codifies the amino acid sequence of proteins. The proteins participate in processes resulting in the traits of the organism and spanning over different levels of biological organization. |  |  |  |  |  |
| 12 | Mutations (changes to the DNA sequences) sometimes have no effect on an organism. |  |  |  |  |  |
| 13 | A gene that has been damaged (for instance by radiation) will definitely lead to cancer. |  |  |  |  |  |

## Section 3: Principles of genomics

| Q. | ***On a scale from strongly disagree to strongly agree, to what extent do you agree with the following statements?***  *(NB: Please only chose “don’t know” if you do not understand the statement)* | Strongly disagree | Disagree | Agree | Strongly agree | Don’t know |
| --- | --- | --- | --- | --- | --- | --- |
| 14 | The genome consists of all organism’s genes that code for the production of proteins. |  |  |  |  |  |
| 15 | Only a small proportion of the human genome consists of genes that code for the production of proteins. |  |  |  |  |  |
| 16 | Most of the human genome consists of genes that code for the production of proteins. |  |  |  |  |  |
| 17 | The human genome contains more genes than the genome of any other living being. |  |  |  |  |  |
| 18 | Humans have about the same amount of genes as a fruit fly (between 20,000 and 30,000 genes). |  |  |  |  |  |
| 19 | The human genome has fewer genes than some less complex organisms such as tomato plants and rice. |  |  |  |  |  |
| 20 | Cells, tissues and organs differ because they have different sets of genes that are activated (“turned on”) and deactivated (“turned off”). |  |  |  |  |  |
| 21 | Only cells in the eyes have genetic information for eye colour. |  |  |  |  |  |
| 22 | Every cell of the body contains the whole genome. |  |  |  |  |  |
| 23 | Genes can be activated or deactivated by other genes. |  |  |  |  |  |
| 24 | If a cell lacks a certain substance, such as a vitamin, a gene can be deactivated. |  |  |  |  |  |
| 25 | Smoking has no effect on gene activity. |  |  |  |  |  |
| 26 | When someone says something is “epigenetic” it means that you can inherit changes in gene activity without inheriting changes in the DNA sequence. |  |  |  |  |  |
| 27 | When someone says something is “epigenetic”, it means that environmental factors can change part of the DNA sequence |  |  |  |  |  |
| 28 | When someone talks of an epigenetic change she is referring to a large change in the DNA sequence. |  |  |  |  |  |
| 29 | Diet can cause changes in gene activity that are passed down through generations. |  |  |  |  |  |
| 30 | Epigenetic changes are not influenced by environmental factors. |  |  |  |  |  |
| 31 | Epigenetic changes are triggered by mutations. |  |  |  |  |  |

# Section 4: Attitudes towards applications of genetics and genomics

## Gene therapy

Gene therapy involves the replacement of a faulty gene in an affected tissue in the body by a normally-functioning gene. Research is still at the clinical trial stage, but recent advances are offering new hope for future treatment in humans.

| Q. | ***On a scale from strongly disagree to strongly agree, to what extent do you agree with the following statements?***  *(NB: Please only chose “don’t know” if you do not understand the statement)* | Strongly disagree | Disagree | Agree | Strongly agree | Don’t know |
| --- | --- | --- | --- | --- | --- | --- |
| 32 | I am skeptical toward gene therapy because I am scared by the thought of interfering with our genes |  |  |  |  |  |
| 33 | If I had a serious genetic disorder I would consider undergoing gene therapy to try to cure it. |  |  |  |  |  |
| 34 | I would be glad if gene therapy was available for people with serious genetic disorders. |  |  |  |  |  |
| 35 | I worry about gene therapy being used for modify or enhance physical attributes such as athletic performance. |  |  |  |  |  |
| 36 | I am generally positive towards gene therapy and think the government should invest more money into its development. |  |  |  |  |  |

## Genetic testing

A genetic test examines your DNA, and can reveal changes or variations in your genes that may be associated with an illness or a disorder. A genetic test can be arranged by your doctor or health clinic, or in some countries you can order a genetic test yourself on the internet (called “direct-to-consumer” testing).

| Q. | ***On a scale from strongly disagree to strongly agree, to what extent do you agree with the following statements?***  *(NB: Please only chose “don’t know” if you do not understand the statement)* | Strongly disagree | Disagree | Agree | Strongly agree | Don’t know |
| --- | --- | --- | --- | --- | --- | --- |
| 37 | At some point in my life, I might consider having a genetic test to find out my risk of developing various genetic diseases. |  |  |  |  |  |
| 38 | I am skeptical towards genetic tests that can be ordered on the internet because it may be difficult to interpret the results correctly. |  |  |  |  |  |
| 39 | I am glad that genetic tests are available for people with a family history of serious genetic disease to find out if they are at risk. |  |  |  |  |  |
| 40 | I worry about the possibility that the results of genetic tests get into the hands of insurance companies or future employers. |  |  |  |  |  |
| 41 | I am generally positive towards genetic testing and think the government should invest more money into its development. |  |  |  |  |  |

## Prenatal genetic testing

Prenatal genetic diagnosis is used to detect changes or variations in a fetus’s genes or chromosomes before birth, to see if it has any chromosomal or genetic defects. This can help parents know more about the future possibilities in the life of their children.

| Q. | ***On a scale from strongly disagree to strongly agree, to what extent do you agree with the following statements?***  *(NB: Please only chose “don’t know” if you do not understand the statement)* | Strongly disagree | Disagree | Agree | Strongly agree | Don’t know |
| --- | --- | --- | --- | --- | --- | --- |
| 42 | If I had a family history of a serious genetic disease, I would definitely want to use prenatal genetic diagnosis. |  |  |  |  |  |
| 43 | I do not think prenatal diagnosis should be made available for detecting conditions such as asthma and Attention Deficit Hyperactivity Disorder (ADHD). |  |  |  |  |  |
| 44 | The government should make prenatal genetic testing available to all individuals who want it. |  |  |  |  |  |
| 45 | Prenatal genetic testing should not be allowed, unless for exceptional cases of severe genetic disease in a family. |  |  |  |  |  |
| 46 | I am generally positive towards prenatal genetic diagnosis and think the government should invest more money into its development. |  |  |  |  |  |

## Personalised medicine and pharmacogenomics

Genetic testing is the first step in personalized medicine and pharmacogenomics. Personalised medicine uses knowledge of a person’s genes to predict his or her risk for developing a particular disease and to influence decisions about lifestyle in order to help prevent or manage a disease. Pharmacogenomics uses genetic information to find the best medicine or treatment for a disease, for example when doctors predict how an individual’s genes will respond to certain cancer drugs. In some cases doctors analyze particular genes associated with the cancer, and in other cases they may analyze the entire genome of the individual.

| Q. | ***On a scale from strongly disagree to strongly agree, to what extent do you agree with the following statements?***  *(NB: Please only chose “don’t know” if you do not understand the statement)* | Strongly disagree | Disagree | Agree | Strongly agree | Don’t know |
| --- | --- | --- | --- | --- | --- | --- |
| 47 | If I were diagnosed with cancer, I would consider getting my genes analysed in order to help chose a cancer treatment with the fewest side effects. |  |  |  |  |  |
| 48 | If I had a family history of diabetes I would consider getting my genes analysed in order to help me make lifestyle choices and decisions about interventions that may prevent diabetes from developing. |  |  |  |  |  |
| 49 | I would not be willing to get my whole genome analysed, because I worry about issues of confidentiality. |  |  |  |  |  |
| 50 | I am sceptical toward pharmacogenomics because of the possibility of getting clinical information about my genes that is unrelated to the treatment with certain drugs. |  |  |  |  |  |
| 51 | I am generally positive towards personalized medicine and pharmacogenomics and think the government should invest more money into its development. |  |  |  |  |  |
