# Supplementary material for: Young Adults’ Belief in Genetic Determinism, and Knowledge and Attitudes towards Modern Genetics and Genomics: The PUGGS Questionnaire
Source: PLoS One. 2017 Jan 23;12(1):e0169808. doi: 10.1371/journal.pone.0169808 (PMC5256916; doi:10.1371/journal.pone.0169808)
Supplement: S4 Table — (DOCX) [file pone.0169808.s004.docx]

Supporting Information 5

Code Book used in the first pilot study of the PUGGS questionnaire

Section 1 - Background information:

| **Variable** | **Question** | **Primary code** | **Secondary code** |
| --- | --- | --- | --- |
| **Age** | i. What is your age group? | 1=15 or younger  2=16-18  3=19-21  4=22 04 older | N/A (not applicable) |
| **Gender** | ii. What is your gender? | 1=male  2=female  3=other | N/A |
| **Field of study** | iii. What is your main field of study? | 1=science & technology  2=humanities  3=health  4=arts  5=other | N/A |
| **Religion** | iv. Are your opinions influenced by religion | 1=greatly influenced  2=somewhat influenced  3=not influenced at all | N/A |
| **Experience with genetics** | v. Have you or anyone close to you ever had any experience with genetic issues? | 1=yes  2=no | N/A |

Section 2: The influence of genes and environment on traits

Part 1 - Table of Traits:

Measuring: Degree of determinism

| Variable/Question | Primary code | | Expected answer |
| --- | --- | --- | --- |
| TT1 (coronary heart disease) | | 1=only environmental  2=largely environmental  3=both  4=largely genetic  5=only genetic  6=don’t know | 3 |
| TT2 (height) | | 1-6, as above | 4 |
| TT3 (bipolar disorder) | | 1-6, as above | 4 |
| TT4 (diabetes) | | 1-6, as above | 2 |
| TT5 (colour blindness) | | 1-6, as above | 5 |
| TT6 (Schizophrenia) | | 1-6, as above | 4 |
| TT7 (Alcoholism) | | 1-6, as above | 3 |
| TT8 (Breast cancer) | | 1-6, as above | 2 |
| TT9 (Interest in fashion) | | 1-6, as above | 1 |
| TT10 (Haemofilia) | | 1-6, as above | 5 |
| TT11 (Addictive gambling behavior) | | 1-6, as above | 2 |
| TT12 (Political beliefs) | | 1-6, as above | 1 |
| TT13 (Intelligence in adults) | | 1-6, as above | 4 |
| TT14 (Major depression) | | 1-6, as above | 3 |
| TT15 (Tourettes syndrome) | | 1-6, as above | 4 |
| TT16 (ADHD) | | 1-6, as above | 4 |
| TT17 (Asthma) | | 1-6, as above | 4 |
| TT18 (Violent behavior) | | 1-6, as above | 2 |
| TT19 (Religious beliefs) | | 1-6, as above | 1 |
| TT20 (Blood group ABO) | | 1-6, as above | 5 |

Section 2 Part 2 (questions 1 to 13)

Measuring: Degree of determinism

| Variable/Question | Primary code | Secondary code (“det”) |
| --- | --- | --- |
| 1. A gene contains the information that codes directly for a trait such as height, eye colour or colour blindness. *(false) - core idea E* | 1=Strongly disagree  2=disagree  3=agree  4=strongly agree  5=don’t know | = 1 (strongly opposing determinism)  = 2 (opposing determinism)  = 3 (favouring determinism)  = 4 (strongly favouring determinism) |
| 2. The majority of human traits and diseases are caused by a single gene. *(false) – core idea A* | 1=Strongly disagree  2=disagree  3=agree  4=strongly agree  5=don’t know | =1 (strongly opposing determinism)  = 2 (opposing determinism)  = 3 (favouring determinism)  = 4 (strongly favouring determinism) |
| 3. Eating habits and physical exercise can play an important role in preventing and controlling diabetes. *(true) – core idea D* | 1=Strongly disagree  2=disagree  3=agree  4=strongly agree  5=don’t know | = 4 (strongly favouring determinism)  = 3 (favouring determinism)  = 2 (opposing determinism  = 1 (strongly opposing determinism) |
| 4. A single gene can influence several different health problems. *(True) – core idea B* | 1=Strongly disagree  2=disagree  3=agree  4=strongly agree  5=don’t know | = 4 (strongly favouring determinism)  = 3 (favouring determinism)  = 2 (opposing determinism  = 1 (strongly opposing determinism) |
| 5. Alzheimer’s disease is influenced by one gene only. *(false) – core idea C* | 1=Strongly disagree  2=disagree  3=agree  4=strongly agree  5=don’t know | = 1 (strongly opposing determinism)  = 2 (opposing determinism)  = 3 (favouring determinism)  = 4 (strongly favouring determinism) |
| 6. In some cases, mutations (changes to the DNA sequences) have no effect on an organism. *(true) – core idea F* | 1=Strongly disagree  2=disagree  3=agree  4=strongly agree  5=don’t know | = 4 (strongly favouring determinism)  = 3 (favouring determinism)  = 2 (opposing determinism  = 1 (strongly opposing determinism) |
| 7. Traits and diseases caused by a single gene are not very common. *(true) – core idea A* | 1=Strongly disagree  2=disagree  3=agree  4=strongly agree  5=don’t know | = 4 (strongly favouring determinism)  = 3 (favouring determinism)  = 2 (opposing determinism  = 1 (strongly opposing determinism) |
| 8. Personality is caused by genes only. *(false) – core idea D* | 1=Strongly disagree  2=disagree  3=agree  4=strongly agree  5=don’t know | = 1 (strongly opposing determinism)  = 2 (opposing determinism)  = 3 (favouring determinism)  = 4 (strongly favouring determinism) |
| 9. A gene contains the information that codes for the amino acid sequence of proteins. The proteins participate in processes that result in the organism’s traits, spanning over different levels of biological organization. *(true) – core idea E* | 1=Strongly disagree  2=disagree  3=agree  4=strongly agree  5=don’t know | = 4 (strongly favouring determinism)  = 3 (favouring determinism)  = 2 (opposing determinism  = 1 (strongly opposing determinism) |
| 10. A gene can only influence a single health problem. *(False)- core idea B* | 1=Strongly disagree  2=disagree  3=agree  4=strongly agree  5=don’t know | = 1 (strongly opposing determinism)  = 2 (opposing determinism)  = 3 (favouring determinism)  = 4 (strongly favouring determinism) |
| 11. Most traits and diseases are caused by both genes and environmental factors. *(true) – core idea D* | 1=Strongly disagree  2=disagree  3=agree  4=strongly agree  5=don’t know | = 4 (strongly favouring determinism)  = 3 (favouring determinism)  = 2 (opposing determinism  = 1 (strongly opposing determinism) |
| 12. Intelligence is influenced by many different genes. *(true) – core idea C* | 1=Strongly disagree  2=disagree  3=agree  4=strongly agree  5=don’t know | = 4 (strongly favouring determinism)  = 3 (favouring determinism)  = 2 (opposing determinism  = 1 (strongly opposing determinism) |
| 13. A gene that has been damaged (by radiation, for example) will definitely lead to cancer. *(false) – core idea F* | 1=Strongly disagree  2=disagree  3=agree  4=strongly agree  5=don’t know | = 1 (strongly opposing determinism)  = 2 (opposing determinism)  = 3 (favouring determinism)  = 4 (strongly favouring determinism) |
|  |  |  |

Section 3: Principles of genomics

Measuring: Understanding of genomics

| Variable/Question | Primary code | Secondary code (“und”) |
| --- | --- | --- |
| 14. The genome consists of all the genes in an organism that code for the production of proteins. *(false) – core idea G* | 1=Strongly disagree  2=disagree  3=agree  4=strongly agree  5=don’t know | = 4 (full understanding)  = 3 (some understanding)  = 2 (some misunderstanding)  = 1 (complete misunderstanding) |
| 15. Cells, tissues and organs differ because they have different sets of genes that are activated (“turned on”) and deactivated (“turned off”). *(true) – core idea I* | 1=Strongly disagree  2=disagree  3=agree  4=strongly agree  5=don’t know | = 1 (complete misunderstanding)  = 2 (some misunderstanding)  = 3 (some understanding)  = 4 (full understanding) |
| 16. Environmental factors, such as cigarette smoke, has no effect on gene activity. *(false) – core idea J* | 1=Strongly disagree  2=disagree  3=agree  4=strongly agree  5=don’t know | = 4 (full understanding)  = 3 (some understanding)  = 2 (some misunderstanding)  = 1 (complete misunderstanding) |
| 17. The human genome contains more genes than the genome of any other living being. *(false) – core idea H* | 1=Strongly disagree  2=disagree  3=agree  4=strongly agree  5=don’t know | = 4 (full understanding)  = 3 (some understanding)  = 2 (some misunderstanding)  = 1 (complete misunderstanding) |
| 18. Every cell of the body contains the whole genome.  *(true) – core idea I* | 1=Strongly disagree  2=disagree  3=agree  4=strongly agree  5=don’t know | = 1 (complete misunderstanding)  = 2 (some misunderstanding)  = 3 (some understanding)  = 4 (full understanding) |
| 19. When someone says something is “epigenetic” it means that you can inherit changes in gene activity without inheriting changes in the DNA sequence. *(true) – core idea K* | 1=Strongly disagree  2=disagree  3=agree  4=strongly agree  5=don’t know | = 1 (complete misunderstanding)  = 2 (some misunderstanding)  = 3 (some understanding)  = 4 (full understanding) |
| 20. Only a small proportion of the human genome consists of genes that code for the production of proteins.  *(true) – core idea G* | 1=Strongly disagree  2=disagree  3=agree  4=strongly agree  5=don’t know | = 1 (complete misunderstanding)  = 2 (some misunderstanding)  = 3 (some understanding)  = 4 (full understanding) |
| 21. Epigenetic changes are not influenced by environmental factors.  *(false) – core idea L* | 1=Strongly disagree  2=disagree  3=agree  4=strongly agree  5=don’t know | = 4 (full understanding)  = 3 (some understanding)  = 2 (some misunderstanding)  = 1 (complete misunderstanding) |
| 22. Humans have about the same amount of genes as a fruit fly (between 20,000 and 30,000 genes).  *(true) – core idea H* | 1=Strongly disagree  2=disagree  3=agree  4=strongly agree  5=don’t know | = 1 (complete misunderstanding)  = 2 (some misunderstanding)  = 3 (some understanding)  = 4 (full understanding) |
| 23. When someone talks of an epigenetic change she is referring to a large change in the DNA sequence.  *(false) – core idea K* | 1=Strongly disagree  2=disagree  3=agree  4=strongly agree  5=don’t know | = 4 (full understanding)  = 3 (some understanding)  = 2 (some misunderstanding)  = 1 (complete misunderstanding) |
| 24. Epigenetic changes are caused by mutations.  *(false) – core idea L* | 1=Strongly disagree  2=disagree  3=agree  4=strongly agree  5=don’t know | = 4 (full understanding)  = 3 (some understanding)  = 2 (some misunderstanding)  = 1 (complete misunderstanding) |
| 25. Most of the human genome consists of genes that code for the production of proteins.  *(false) – core idea G* | 1=Strongly disagree  2=disagree  3=agree  4=strongly agree  5=don’t know | = 4 (full understanding)  = 3 (some understanding)  = 2 (some misunderstanding)  = 1 (complete misunderstanding) |
| 26. The human genome has fewer genes than some less complex organisms such as tomato plants and rice. *(true) – core idea H* | 1=Strongly disagree  2=disagree  3=agree  4=strongly agree  5=don’t know | = 1 (complete misunderstanding)  = 2 (some misunderstanding)  = 3 (some understanding)  = 4 (full understanding) |
| 27. When someone says something is “epigenetic”, it means that environmental factors can change part of the DNA sequence.  *(false) – core idea K* | 1=Strongly disagree  2=disagree  3=agree  4=strongly agree  5=don’t know | = 4 (full understanding)  = 3 (some understanding)  = 2 (some misunderstanding)  = 1 (complete misunderstanding) |
| 28. Genes can be activated or deactivated by other genes.  *(true) – core idea J* | 1=Strongly disagree  2=disagree  3=agree  4=strongly agree  5=don’t know | = 1 (complete misunderstanding)  = 2 (some misunderstanding)  = 3 (some understanding)  = 4 (full understanding) |
| 29. Eating habits can cause changes in gene activity that are passed down from generation to generation. *(true) – core idea L* | 1=Strongly disagree  2=disagree  3=agree  4=strongly agree  5=don’t know | = 1 (complete misunderstanding)  = 2 (some misunderstanding)  = 3 (some understanding)  = 4 (full understanding) |
| 30. Only eye cells have genetic information for eye colour. *(false) – core idea I* | 1=Strongly disagree  2=disagree  3=agree  4=strongly agree  5=don’t know | = 4 (full understanding)  = 3 (some understanding)  = 2 (some misunderstanding)  = 1 (complete misunderstanding) |
| 31. If a cell lacks a certain substance, such as a vitamin, a gene can be deactivated.  *(true) – core idea J* | 1=Strongly disagree  2=disagree  3=agree  4=strongly agree  5=don’t know | = 1 (complete misunderstanding)  = 2 (some misunderstanding)  = 3 (some understanding)  = 4 (full understanding) |

Section 4: Attitudes

Measuring: Positive or negative attitude

| Variable/Question | Primary code | Secondary code (“att”) |
| --- | --- | --- |
| 32. I am skeptical toward gene therapy because I am scared by the thought of interfering with our genes. | 1=Strongly disagree  2=disagree  3=agree  4=strongly agree  5=don’t know | = 4 (very positive)  = 3 (fairly positive)  = 2 (fairly negative)  = 1 (very negative) |
| 33. If I had a serious genetic disorder I would consider undergoing gene therapy to try to cure it. | 1=Strongly disagree  2=disagree  3=agree  4=strongly agree  5=don’t know | = 1 (very negative)  = 2 (fairly negative)  = 3 (fairly positive)  = 4 (very positive) |
| 34. I would be glad if gene therapy was available for people with serious genetic disorders. | 1=Strongly disagree  2=disagree  3=agree  4=strongly agree  5=don’t know | = 1 (very negative)  = 2 (fairly negative)  = 3 (fairly positive)  = 4 (very positive) |
| 35. I would be worried about gene therapy being used to modify or enhance physical attributes such as athletic performance. | 1=Strongly disagree  2=disagree  3=agree  4=strongly agree  5=don’t know | = 4 (very positive)  = 3 (fairly positive)  = 2 (fairly negative)  = 1 (very negative) |
| 36. I am generally positive towards gene therapy and think the government should invest more money into its development. | 1=Strongly disagree  2=disagree  3=agree  4=strongly agree  5=don’t know | = 1 (very negative)  = 2 (fairly negative)  = 3 (fairly positive)  = 4 (very positive) |
| 37. At some point in my life, I might consider having a genetic test to find out my risk of developing various genetic diseases. | 1=Strongly disagree  2=disagree  3=agree  4=strongly agree  5=don’t know | = 1 (very negative)  = 2 (fairly negative)  = 3 (fairly positive)  = 4 (very positive) |
| 38. I am sceptical towards genetic tests that can be ordered on the internet because it may be difficult to interpret the results correctly. | 1=Strongly disagree  2=disagree  3=agree  4=strongly agree  5=don’t know | = 4 (very positive)  = 3 (fairly positive)  = 2 (fairly negative)  = 1 (very negative) |
| 39. I am glad that genetic tests are available so that people with a family history of serious genetic disease can find out if they are at risk. | 1=Strongly disagree  2=disagree  3=agree  4=strongly agree  5=don’t know | = 1 (very negative)  = 2 (fairly negative)  = 3 (fairly positive)  = 4 (very positive) |
| 40. I worry about the possibility that the results of genetic tests get into the hands of insurance companies or future employers. | 1=Strongly disagree  2=disagree  3=agree  4=strongly agree  5=don’t know | = 4 (very positive)  = 3 (fairly positive)  = 2 (fairly negative)  = 1 (very negative) |
| 41. I am generally positive towards genetic testing and think the government should invest more money into its development. | 1=Strongly disagree  2=disagree  3=agree  4=strongly agree  5=don’t know | = 1 (very negative)  = 2 (fairly negative)  = 3 (fairly positive)  = 4 (very positive) |
| 42. If I had a family history of a serious genetic disease, I would definitely want to use prenatal genetic diagnosis. | 1=Strongly disagree  2=disagree  3=agree  4=strongly agree  5=don’t know | = 1 (very negative)  = 2 (fairly negative)  = 3 (fairly positive)  = 4 (very positive) |
| 43. I do not think prenatal diagnosis should be made available for detecting conditions such as asthma and Attention Deficit Hyperactivity Disorder (ADHD). | 1=Strongly disagree  2=disagree  3=agree  4=strongly agree  5=don’t know | = 4 (very positive)  = 3 (fairly positive)  = 2 (fairly negative)  = 1 (very negative) |
| 44. The government should make prenatal genetic testing available to all individuals who want it. | 1=Strongly disagree  2=disagree  3=agree  4=strongly agree  5=don’t know | = 1 (very negative)  = 2 (fairly negative)  = 3 (fairly positive)  = 4 (very positive) |
| 45. Prenatal genetic testing should not be allowed, unless for exceptional cases of severe genetic disease in a family. | 1=Strongly disagree  2=disagree  3=agree  4=strongly agree  5=don’t know | = 4 (very positive)  = 3 (fairly positive)  = 2 (fairly negative)  = 1 (very negative) |
| 46. I am generally positive towards prenatal genetic diagnosis and think the government should invest more money into its development. | 1=Strongly disagree  2=disagree  3=agree  4=strongly agree  5=don’t know | = 1 (very negative)  = 2 (fairly negative)  = 3 (fairly positive)  = 4 (very positive) |
| 47. If I were diagnosed with cancer, I would consider having my genes analysed in order to help chose a cancer treatment with the fewest side effects. | 1=Strongly disagree  2=disagree  3=agree  4=strongly agree  5=don’t know | = 1 (very negative)  = 2 (fairly negative)  = 3 (fairly positive)  = 4 (very positive) |
| 48. If I had a family history of diabetes I would consider having my genes analysed in order to help me make lifestyle choices and decisions about interventions that may prevent diabetes from developing. | 1=Strongly disagree  2=disagree  3=agree  4=strongly agree  5=don’t know | = 1 (very negative)  = 2 (fairly negative)  = 3 (fairly positive)  = 4 (very positive) |
| 49. I would not be willing to get my whole genome analysed, because I worry about issues of confidentiality. | 1=Strongly disagree  2=disagree  3=agree  4=strongly agree  5=don’t know | = 4 (very positive)  = 3 (fairly positive)  = 2 (fairly negative)  = 1 (very negative) |
| 50. I am sceptical toward pharmacogenomics because of the possibility of getting information about my genes that is unrelated to the treatment. | 1=Strongly disagree  2=disagree  3=agree  4=strongly agree  5=don’t know | = 4 (very positive)  = 3 (fairly positive)  = 2 (fairly negative)  = 1 (very negative) |
| 51. I am generally positive towards personalized medicine and pharmacogenomics and think the government should invest more money into its development. | 1=Strongly disagree  2=disagree  3=agree  4=strongly agree  5=don’t know | = 1 (very negative)  = 2 (fairly negative)  = 3 (fairly positive)  = 4 (very positive) |
