# Supplementary material for: Young Adults’ Belief in Genetic Determinism, and Knowledge and Attitudes towards Modern Genetics and Genomics: The PUGGS Questionnaire
Source: PLoS One. 2017 Jan 23;12(1):e0169808. doi: 10.1371/journal.pone.0169808 (PMC5256916; doi:10.1371/journal.pone.0169808)
Supplement: S1 Text — (DOCX) [file pone.0169808.s007.docx]

# S3 Informed consent form


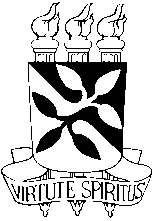


PUBLIC UNDERSTANDING AND ATTITUDES TOWARDS CONTEMPORARY GENETICS AND NEW GENETIC TECHNOLOGIES

Dear student,

You are being invited to collaborate with a study conducted by the lab [to be completed after reviewing], in which the researchers intend to investigate public understanding about contemporary genetics and genetic technologies. We hope the knowledge constructed through this study can contribute to changes in curricula, textbooks, and ways of teaching and communicating about genetics topics.

Your participation is very important and consists in answering a questionnaire. Some students may be selected to give their opinion about the questions and other aspects of the instrument. It is important to highlight that this is not an evaluation, will not be used for grading, and the questionnaire is confidential and does not identify the student. Only the university researchers will have acess to the answers. We are interested in your opinions, not in testing your knowledge.

We ask you to read the following consent form and give your consent to participate in the study, if you wish.

We count on your participation!

**INFORMED CONSENT FORM**

**Title of the study**: Public understanding and atitudes towards contemporary genetics and new genetic technologies.

1. **Nature of the study**: I, ____________________________________________, am being invited to participate in a study entitled “Public understanding and atitudes towards contemporary genetics and new genetic technologies”, which has the goal of investigating the understanding and atitudes of the general public towards genetics and genetic technologies.
2. **Involvement in the study**: to participate in this study, I will answer a questionnaire. I am aware that I am free to refuse to participate and eventually refuse to participate at any stage of the study, with no harm. Whenever I want I can ask for more information about the study through the phone numbers of the researchers.
3. **About the questionnaire:** I am aware that the questionnaire contains questions about genes and their relations to traits, as well as about genetic technologies, besides some personal data like age, gender, etc.
4. **Risk and discomfort**: the participation in this study does not bring legal problems. The procedures used in the study conform to the Ethical Criteria to Research with Human Beings, according to Resolution n^o.^ 466 from the Health Research Council, from December 12^th^ 2012. None of these procedures offers any risk to my dignity.
5. **Confidentiality**: all the information gathered in this study is strictly confidential. Only the researchers will have knowledge of the data.
6. **Benefits**: I am aware that, by participating in this study, I will not receive any direct benefit. However, is expected that this study brings important information about public understanding of contemporary genetics and attitudes towards genetic technologies. Thus, is is expected that the knowledge resulting from this study can contribute to changes in curricula, textbooks, and the way of teaching and communicating about genetics topics. The researchers are committed to publicizing the obtained results so as to potentialize social and educational positive effects.
7. **Payment**: I will not have any expense to participate in the study, as well as nothing will be paid for my participation.
8. **Publication**: I am aware of and authorize the publication of the data gathered through the questionnaire in scientific journals, provided confidentiality is respected.

This informed consent form will be emitted in duplicate, one of each will be kept by the participant, the other by the researchers.

After being informed about the content of the study and understanding its nature and goals, I manifest my free consent to participate in it. I declare I received a copy of this signed informed consent and authorize the use of the data obtained through my participation in this study, always respecting my confidentiality

.

Salvador, _____, de ______________ de 20____.

________________________________________________

Participante signature

__________________________________

Charbel Niño El-Hani

__________________________________

Neima Alice Menezes Evangelista

__________________________________

Rebecca Bruu Carver
