# Supplementary material for: Young Adults’ Belief in Genetic Determinism, and Knowledge and Attitudes towards Modern Genetics and Genomics: The PUGGS Questionnaire
Source: PLoS One. 2017 Jan 23;12(1):e0169808. doi: 10.1371/journal.pone.0169808 (PMC5256916; doi:10.1371/journal.pone.0169808)
Supplement: S2 Text — (DOCX) [file pone.0169808.s008.docx]

Supporting Information 6

Code Book used in the second pilot study of the PUGGS questionnaire

# Section 1: Basic information

i. Age

1 = 15 or younger

2 = 16-18 years old

3 = 19-21 years old

4 = 22 or older

ii. Gender

1 = male

2 = female

3 = other (transgender, transsexual)

iii. Field of study

1 = Science and technology

2 = Humanities

3 = Health

4 = Arts

5 = Other

iv. Year (manual)

v. Religion

1 = greatly influenced

2 = somewhat influenced

3 = not influenced

vi. Experience with genetic issues

1 = yes

2 = no

# Section 2 (Belief in determinism/table of traits)

In general for this section:

1 = Only environmental differences

2 = Mainly environmental differences

3 = Both genetic and environmental differences equal

4 = Mainly genetic differences

5 = Only genetic differences

99 = missing

# Section 3 (Determinism questions/gene-env. interaction)

Primary codes:

1 = True

2 = False

3 = don’t know

99 = missing

Secondary codes:

0 = incorrect or don’t know or missing

1 = correct

Question 1 (Q1)

If answer = 1 (true) = 0 (incorrect)

If answer = 2 (false) = 1 (correct)

If answer = 3 (don't know) = 0

If answer = 99 = 0

Q2

If answer 1 = 0

If answer 2 (false) = 1 (correct)

If answer 3 = 0

If answer 99 = 0

Q3

If answer 1 (true) = 1 (correct)

If answer 2 = 0

If answer 3 = 0

If answer 99 = 0

Q4

If answer 1 = 0

If answer 2 (false) = 1 (correct)

If answer 3 = 0

If answer 99 = 0

Q5

If answer 1 (true) = 1 (correct)

If answer 2 = 0

If answer 3 = 0

If answer 99 = 0

Q6

If answer 1 = 0

If answer 2 (false) = 1 (correct)

If answer 3 = 0

If answer 99 = 0

Q7

If answer 1 = 0

If answer 2 (false) = 1 (correct)

If answer 3 = 0

If answer 99 = 0

Q8

If answer 1 (true) = 1 (correct)

If answer 2 = 0

If answer 3 = 0

If answer 99 = 0

Q9

If answer 1 (true) = 1 (correct)

If answer 2 = 0

If answer 3 = 0

If answer 99 = 0

# Section 4: Knowledge about modern genetics an genomics

Primary codes:

1 = True

2 = False

3 = don’t know

99 = missing

Secondary codes:

0 = incorrect or don’t know or missing

1 = correct

Q10

If answer 1 = 0

If answer 2 (false) = 1 (correct)

If answer 3 = 0

If answer 99 = 0

Q11

If answer 1 (true) = 1 (correct)

If answer 2 = 0

If answer 3 = 0

If answer 99 = 0

Q12

If answer 1 (true) = 1 (correct)

If answer 2 = 0

If answer 3 = 0

If answer 99 = 0

Q13

If answer 1 = 0

If answer 2 (false) = 1 (correct)

If answer 3 = 0

If answer 99 = 0

Q14

If answer 1 (true) = 1 (correct)

If answer 2 = 0

If answer 3 = 0

If answer 99 = 0

Q15

If answer 1 (true) = 1 (correct)

If answer 2 = 0

If answer 3 = 0

If answer 99 = 0

Q16

If answer 1 (true) = 1 (correct)

If answer 2 = 0

If answer 3 = 0

If answer 99 = 0

Q17

If answer 1 (true) = 1 (correct)

If answer 2 = 0

If answer 3 = 0

If answer 99 = 0

Q18

If answer 1 = 0

If answer 2 (false) = 1 (correct)

If answer 3 = 0

If answer 99 = 0

Q19

If answer 1 = 0

If answer 2 (false) = 1 (correct)

If answer 3 = 0

If answer 99 = 0

Q20

If answer 1 = 0

If answer 2 (false) = 1 (correct)

If answer 3 = 0

If answer 99 = 0

Q21

If answer 1 (true) = 1 (correct)

If answer 2 = 0

If answer 3 = 0

If answer 99 = 0

Q22

If answer 1 = 0

If answer 2 (false) = 1 (correct)

If answer 3 = 0

If answer 99 = 0

Q23

If answer 1 (true) = 1 (correct)

If answer 2 = 0

If answer 3 = 0

If answer 99 = 0

Q24

If answer 1 = 0

If answer 2 (false) = 1 (correct)

If answer 3 = 0

If answer 99 = 0

Q25

If answer 1 (true) = 1 (correct)

If answer 2 = 0

If answer 3 = 0

If answer 99 = 0

# Section 5: Attitudes

Primary codes:

1 = strongly disagree

2 = disagree

3 = agree

4 = strongly agree

Secondary codes:

1 = very negative

2 = fairly negative

3 = fairly positive

4 = very positive

**Gene therapy:**

Q26 (reversed coding)

If answer is 1 = 4 (very positive)

If answer is 2 = 3 (fairly positive)

If answer is 3 = 2 (fairly negative)

If answer is 4 = 1 (very negative)

Q27

If answer is 1 = 1 (very negative)

If answer is 2 = 2 (fairly negative)

If answer is 3 = 3 (fairly positive)

If answer is 4 = 4 (very positive)

Q28

If answer is 1 = 1 (very negative)

If answer is 2 = 2 (fairly negative)

If answer is 3 = 3 (fairly positive)

If answer is 4 = 4 (very positive)

Q29

If answer is 1 = 1 (very negative)

If answer is 2 = 2 (fairly negative)

If answer is 3 = 3 (fairly positive)

If answer is 4 = 4 (very positive)

Q30

If answer is 1 = 1 (very negative)

If answer is 2 = 2 (fairly negative)

If answer is 3 = 3 (fairly positive)

If answer is 4 = 4 (very positive)

**Gene therapy:**

Q31

If answer is 1 = 1 (very negative)

If answer is 2 = 2 (fairly negative)

If answer is 3 = 3 (fairly positive)

If answer is 4 = 4 (very positive)

Q32

If answer is 1 = 1 (very negative)

If answer is 2 = 2 (fairly negative)

If answer is 3 = 3 (fairly positive)

If answer is 4 = 4 (very positive)

Q33

If answer is 1 = 1 (very negative)

If answer is 2 = 2 (fairly negative)

If answer is 3 = 3 (fairly positive)

If answer is 4 = 4 (very positive)

Q34 (reversed coding)

If answer is 1 = 4 (very positive)

If answer is 2 = 3 (fairly positive)

If answer is 3 = 2 (fairly negative)

If answer is 4 = 1 (very negative)

Q35

If answer is 1 = 1 (very negative)

If answer is 2 = 2 (fairly negative)

If answer is 3 = 3 (fairly positive)

If answer is 4 = 4 (very positive)

**Prenatal genetic testing**

Q36

If answer is 1 = 1 (very negative)

If answer is 2 = 2 (fairly negative)

If answer is 3 = 3 (fairly positive)

If answer is 4 = 4 (very positive)

Q37 (reversed coding)

If answer is 1 = 4 (very positive)

If answer is 2 = 3 (fairly positive)

If answer is 3 = 2 (fairly negative)

If answer is 4 = 1 (very negative)

Q38

If answer is 1 = 1 (very negative)

If answer is 2 = 2 (fairly negative)

If answer is 3 = 3 (fairly positive)

If answer is 4 = 4 (very positive)

Q39 (reversed coding)

If answer is 1 = 4 (very positive)

If answer is 2 = 3 (fairly positive)

If answer is 3 = 2 (fairly negative)

If answer is 4 = 1 (very negative)

Q40

If answer is 1 = 1 (very negative)

If answer is 2 = 2 (fairly negative)

If answer is 3 = 3 (fairly positive)

If answer is 4 = 4 (very positive)

**Personalised medicine and pharmacogenomics**

Q41

If answer is 1 = 1 (very negative)

If answer is 2 = 2 (fairly negative)

If answer is 3 = 3 (fairly positive)

If answer is 4 = 4 (very positive)

Q42

If answer is 1 = 1 (very negative)

If answer is 2 = 2 (fairly negative)

If answer is 3 = 3 (fairly positive)

If answer is 4 = 4 (very positive)

Q43 (reversed coding)

If answer is 1 = 4 (very positive)

If answer is 2 = 3 (fairly positive)

If answer is 3 = 2 (fairly negative)

If answer is 4 = 1 (very negative)

Q44 (reversed coding)

If answer is 1 = 4 (very positive)

If answer is 2 = 3 (fairly positive)

If answer is 3 = 2 (fairly negative)

If answer is 4 = 1 (very negative)

Q45

If answer is 1 = 1 (very negative)

If answer is 2 = 2 (fairly negative)

If answer is 3 = 3 (fairly positive)

If answer is 4 = 4 (very positive)
